# Trim21 depletion alleviates bone loss in osteoporosis via activation of YAP1/β-catenin signaling

**DOI:** 10.1038/s41413-023-00296-3

**Published:** 2023-10-26

**Authors:** Ri-Xu Liu, Rong-He Gu, Zhi-Peng Li, Zhi-Quan Hao, Qin-Xiao Hu, Zhen-Yan Li, Xiao-Gang Wang, Wang Tang, Xiao-He Wang, Yu-Kai Zeng, Zhen-Wei Li, Qiu Dong, Xiao-Feng Zhu, Di Chen, Ke-Wei Zhao, Rong-Hua Zhang, Zhen-Gang Zha, Huan-Tian Zhang

**Affiliations:** 1 Department of Bone and Joint Surgery, the First Affiliated Hospital of Jinan University; Key Laboratory of Regenerative Medicine of Ministry of Education, Jinan University, Guangzhou, 510630 Guangdong China; 2 Department of Orthopedic and Spine Surgery, The First Affiliated Hospital of Guangzhou Medical University, Guangzhou, 510120 Guangdong China; 3 School of Basic Medical Sciences of Guangxi Medical University, the Fifth Affiliated Hospital of Guangxi Medical University, Nanning, 530022 Guangxi China; 4 Key Laboratory of Big Data-Based Precision Medicine, School of Engineering Medicine, Beihang University, 100191 Beijing, China; 5 Guangdong Provincial Key Laboratory of Traditional Chinese Medicine Informatization, College of Pharmacy, Jinan University, Guangzhou, 510630 Guangdong China; 6 Research Center for Computer-aided Drug Discovery, Shenzhen Institute of Advanced Technology, Chinese Academy of Sciences, 518005 Shenzhen, China; 7 Guangzhou Key Laboratory of Chinese Medicine Research on Prevention and Treatment of Osteoporosis, the Third Affiliated Hospital of Guangzhou University of Chinese Medicine, 510375 Guangzhou, China

**Keywords:** Bone, Pathogenesis

## Abstract

Despite the diverse roles of tripartite motif (Trim)-containing proteins in the regulation of autophagy, the innate immune response, and cell differentiation, their roles in skeletal diseases are largely unknown. We recently demonstrated that Trim21 plays a crucial role in regulating osteoblast (OB) differentiation in osteosarcoma. However, how Trim21 contributes to skeletal degenerative disorders, including osteoporosis, remains unknown. First, human and mouse bone specimens were evaluated, and the results showed that Trim21 expression was significantly elevated in bone tissues obtained from osteoporosis patients. Next, we found that global knockout of the *Trim21* gene (KO, *Trim21*^−/−^) resulted in higher bone mass compared to that of the control littermates. We further demonstrated that loss of Trim21 promoted bone formation by enhancing the osteogenic differentiation of bone marrow mesenchymal stem cells (BMSCs) and elevating the activity of OBs; moreover, Trim21 depletion suppressed osteoclast (OC) formation of RAW264.7 cells. In addition, the differentiation of OCs from bone marrow-derived macrophages (BMMs) isolated from *Trim21*^−/−^ and *Ctsk-cre; Trim21*^*f/f*^ mice was largely compromised compared to that of the littermate control mice. Mechanistically, YAP1/β-catenin signaling was identified and demonstrated to be required for the Trim21-mediated osteogenic differentiation of BMSCs. More importantly, the loss of Trim21 prevented ovariectomy (OVX)- and lipopolysaccharide (LPS)-induced bone loss in vivo by orchestrating the coupling of OBs and OCs through YAP1 signaling. Our current study demonstrated that Trim21 is crucial for regulating OB-mediated bone formation and OC-mediated bone resorption, thereby providing a basis for exploring Trim21 as a novel dual-targeting approach for treating osteoporosis and pathological bone loss.

## Introduction

Bone mass and strength throughout the lifespan are jointly regulated by osteoblast (OB)-mediated bone formation and osteoclast (OC)-mediated bone resorption.^1^ However, under certain circumstances, such as aging and metabolic diseases, an imbalance in bone formation relative to bone resorption results in osteoporosis, which is characterized by the deterioration of bone microarchitecture, low bone density, and poor bone strength.^2^ Accordingly, antiresorptive therapies, such as bisphosphonates and denosumab, have become the first-line therapy for osteoporosis.^2,3^ However, recombinant parathyroid hormone (PTH) (teriparatide), the main anabolic agent currently approved for clinical use in osteoporosis, significantly stimulates bone formation.^3,4^ Increasing evidence has suggested that the coupling of OBs and OCs is crucial for the effect of both antiresorptive therapies and bone anabolic therapies.^5,6^ Anti-bone resorption drugs can inhibit both bone resorption and bone formation, while anabolic drugs mainly enhance bone formation and stimulate bone resorption.^5–7^ Therefore, studies aiming to develop dual-action therapy for suppressing bone resorption and promoting bone formation are emerging and promising for the treatment of osteoporosis and, to some extent, coexisting diseases such as osteoarthritis.^8–10^

Tripartite motif (Trim)-containing proteins have increasingly been recognized as key players in the regulation of several fundamental cellular processes, including autophagy, inflammation, and cell differentiation.^11,12^ For instance, Trim38 has been reported to play an essential role in bone remodeling by facilitating OB differentiation while suppressing OC differentiation by negatively regulating transcription factor-κB (NF-κB) activity in both OCs and OBs.^13^ Trim16 promotes OB differentiation of stem cells by decreasing the carboxy terminus of Hsc70-interacting protein-mediated degradation of Runx2 or in an autophagy-dependent manner.^14,15^ Recent reports have shown that Trim21 is involved in the regulation of the differentiation of several types of immune cells.^16^ Our previous studies have demonstrated that Trim21, despite its role in regulating osteosarcoma cell senescence and proliferation,^17,18^ can favor Runx2-dependent OB differentiation of osteosarcoma cells when deficient.^19^ Consistent with this finding, a recent study also demonstrated that Trim21 negatively regulates the osteogenic differentiation of BMSCs.^20^ However, it remains to be determined whether Trim21 plays a role in regulating normal bone homeostasis and possibly contributes to skeletal degenerative diseases such as osteoporosis.

In this study, we first found that the expression of Trim21 was elevated in bone specimens from osteoporosis patients and ovariectomy (OVX)-induced osteoporotic mice. Furthermore, we observed that *Trim21* knockout mice (KO, *Trim21*^−/−^) had a higher bone mass with increased bone formation and a lower number of OCs than their control littermates (*Trim21*^+/+^). YAP1/β-catenin signaling was identified and demonstrated to be activated upon the loss of *Trim21* in BMSCs and in bone specimens from *Trim21* global and conditional knockout mice. Our study provides new evidence that Trim21 is a promising dual-targeting candidate for preventing bone loss and reducing fracture risk in osteoporosis patients.

## Results

### Trim21 expression is increased in osteoporosis patients, and its deficiency elevates bone mass

**Fig. 1:**
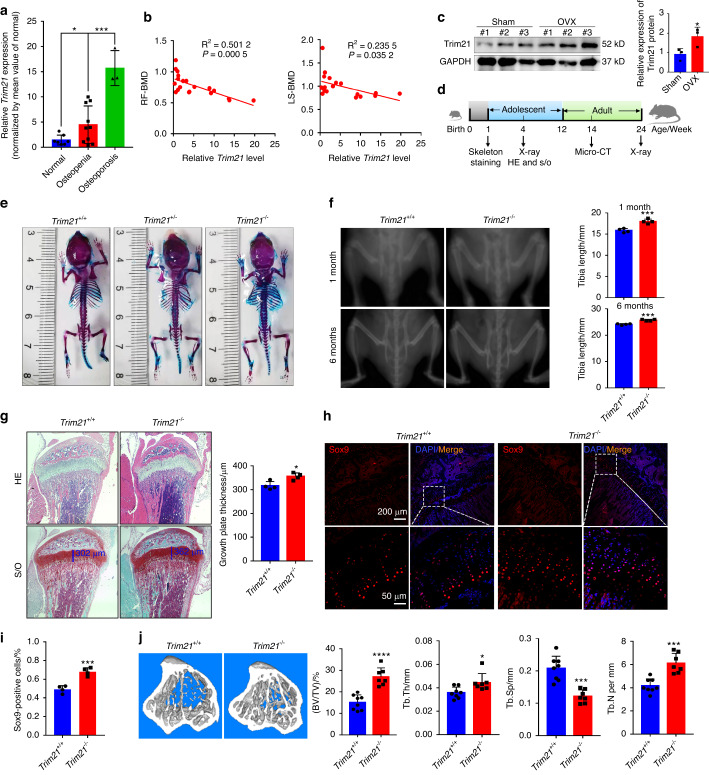
Trim21 is elevated in osteoporotic patients, and its deficiency leads to high bone mass. **a** Quantitative RT‒PCR analysis of *Trim21* mRNA expression in bone specimens from patients with different bone mineral densities (BMDs), which were defined as normal, osteopenia, and osteoporosis. **b** Correlation analysis between *Trim21* mRNA expression and RF-BMD and LS-BMD. RF, right femur; LS, lumbar spine. **c** Immunoblotting analysis of Trim21 protein expression in the lumbar vertebra of 5-month-old sham-operated or ovariectomized mice. **d** Schematic diagram showing the analysis of skeletal parameters of mice at different ages. **e** Alcian blue/Alizarin Red staining of the whole skeleton of 1-week-old *Trim21*^+/+^, *Trim21*^+/−^, and *Trim21*^−/−^ littermates. **f** X-ray images of *Trim21*^*+/+*^ and *Trim21*^−/−^ mice at 1 month and 6 months (left panel). Quantitative analysis of the tibia length of mice at different ages (right panel). **g** Representative H&E and S/O staining images of tibial sections from 1-month-old *Trim21*^+/+^ and *Trim21*^−/−^ mice (left panel). Quantitative analysis of the growth plate thickness of the indicated mice (right panel). **h**, **i** Representative immunofluorescence images (**h**) showing the expression of Sox9^+^ cells (**i**) in growth plates of tibial sections in 1-month-old *Trim21*^+/+^ and *Trim21*^−/−^ mice. **j** Representative micro-CT images of the proximal tibia bone of 14-week-old mice. Quantitative measurements of bone volume per tissue volume (BV/TV), trabecular thickness (Tb. Th), trabecular number (Tb. N), and trabecular separation (Tb.Sp). All bar graphs are presented as the mean ± SD. *P < 0.05; ***P < 0.001; ****P < 0.000 1; n.s. not significant by Student’s *t* test

Trim21 is fundamental for regulating OB differentiation in osteosarcoma,^19^ yet its expression and role in skeletal degenerative disorders, including osteoporosis, are largely unknown. To this end, we first examined the mRNA expression of *Trim21* in bone specimens obtained from patients who underwent joint replacement surgery with different bone mineral densities (BMDs). A total of 20 patients were included in this study, as shown in Table S1. Notably, we found that *Trim21* mRNA levels were increased in patients with osteoporosis (right femur (RF)-BMD T score ≤ −2.5) and osteopenia (RF-BMD T score ≤ −1.0 and > −2.5) compared with those with normal BMDs (T score > −1.0) (Fig. 1a). In addition, a negative correlation was observed between *Trim21* mRNA expression and RF-BMD and lumbar spine (LS)-BMD (Fig. 1b) but not other bone metabolic markers (Fig. S1a–d). A higher expression level of Trim21 was also observed using the LS trabecular bone (TB) of ovariectomized mice compared with that of the sham-operated group (Fig. 1c).

These observations led us to hypothesize that Trim21 plays an important role in regulating bone development and bone mass. Therefore, *Trim21* global knockout (*Trim21*^−/−^) mice were generated and validated by genotyping by reverse transcription polymerase chain reaction (RT‒PCR) (Fig. S2a-c). Next, skeletal staining, X-ray analysis, histological analysis, and micro-CT were used to evaluate the role of Trim21 in dynamic bone modeling in an experimental timeline (Fig. 1d). In accordance with previous reports,^21,22^ *Trim21*^−/−^ mice exhibited no significant difference in body weight and were indistinguishable from their control littermates (*Trim21*^+/+^, wild-type, WT) 7 days after birth, as shown by whole-mount Alcian blue-Alizarin Red S staining (Fig. 1e). Surprisingly, X-ray radiography showed a slight increase in tibia length of the *Trim21*^−/−^ mice compared to the *Trim21*^+/+^ littermates at both 1 month and 6 months of age (Fig. 1f). In addition, similar results were observed in other long bones, such as the femur and humerus, in *Trim21*^−/−^ mice compared to littermate controls (Fig. S3a, b). Moreover, an increase in growth plate thickness was shown by H&E and Safranin O staining (S/O) in 1-month-old *Trim21*^−/−^ mice compared with *Trim21*^+/+^ mice (Fig. 1g).

Sox9 is a transcription factor that plays a critical role in embryogenesis and growth plate formation. Our results revealed that the expression of Sox9 was significantly increased in the long bones of *Trim21*^−/−^ mice compared to their WT littermates (Fig. 1h, i). These findings suggest that Trim21 plays a key role in promoting growth plate proliferation and cartilage development. Next, micro-CT was applied to assess the TB microarchitecture at the proximal tibia of adult (14-week-old) *Trim21*^+/+^ and *Trim21*^−/−^ mice. Notably, *Trim21*^−/−^ mice displayed a greater bone mass in the tibias than *Trim21*^+/+^ mice, as shown by a significant increase in bone volume per tissue volume (BV/TV), trabecular thickness (Tb. Th), and trabecular number (Tb. N) and a decrease in trabecular separation (Tb. Sp) (Fig. 1j). The cortical bone volume was not significantly different between *Trim21*^+/+^ and *Trim21*^−/−^ mice (Fig. S3c). These results collectively suggest that loss of Trim21 results in a high bone mass phenotype in mice.

### Loss of *Trim21* promotes OB differentiation and bone formation

Next, we asked whether Trim21 is required for OB differentiation in mouse BMSCs, calvarial OBs, and MC3T3-E1 cells. After cell expansion, mouse BMSCs were subjected to flow cytometry analysis for surface marker expression (positive for CD29, CD44, CD90, and CD105 but negative for CD34 and CD45) to confirm their undifferentiated status (Fig. S4a). Notably, the protein expression of Trim21 was found to be downregulated during the osteogenic differentiation of BMSCs derived from *Trim21*^+/+^ mice (Fig. S4b, c). Furthermore, the protein and mRNA expression levels of osteogenic markers, including Runx2 and Osterix, were increased in the BMSCs of *Trim21*^−/−^ mice compared to those of control littermates (Fig. S4d–f). Consistently, BMSCs derived from *Trim21*-deficient mice exhibited an increase in osteogenic differentiation, as shown by Alizarin Red S and ALP staining (Fig. S4g, h).

OBs are differentiated from BMSCs, which also give rise to marrow adipocytes; therefore, we examined the possible role of Trim21 in regulating adipocyte differentiation. As expected, the loss of *Trim21* resulted in a significant reduction in the number of Oil Red O-stained cells that were induced by mouse BMSCs, along with suppression of the mRNA expression of adipocyte markers, including *Adipsin*, *Cebpa*, *Fabp4*, and *Pparg* (Fig. S4i). In contrast, overexpression of Trim21 by transient transfection of the plasmid encoding FLAG-Trim21 dramatically abolished OB differentiation (Fig. S4j).

**Fig. 2:**
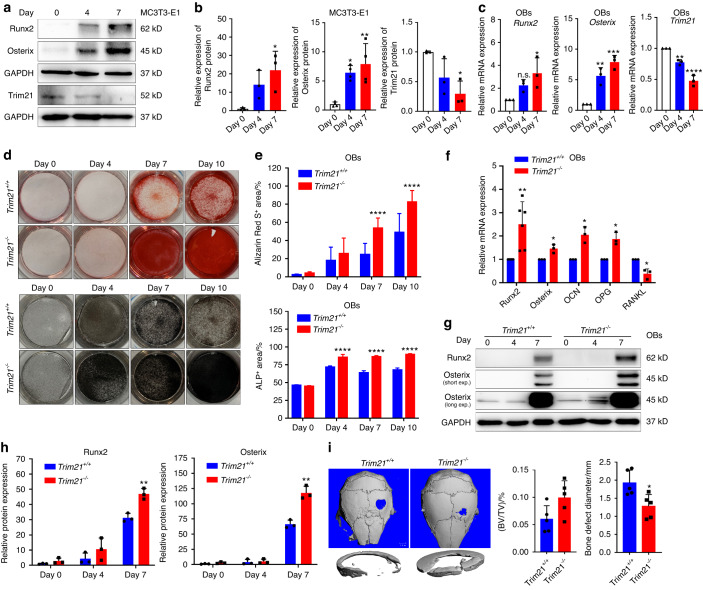
Loss of *Trim21* enhances osteoblast activity and favors bone formation. **a**, **b** Representative immunoblotting analysis (**a**) and quantification of Runx2, Osterix, and Trim21 in MC3T3-E1 cells (**b**) treated with osteogenic medium for 0, 4, and 7 days. **c** Quantitative RT‒PCR analysis of osteogenic biomarker genes (*Osterix, Runx2, and Trim21*) in OBs with osteogenic induction. **d**, **e** Alizarin Red S (upper panel) and ALP (lower panel) staining of primary osteoblasts (OBs) after induction with osteogenic medium for different times (**d**). The percentage of Alizarin Red S- (*n* ≥ 3) and ALP- (*n* ≥ 3) stained area (**e**). **f** Quantitative RT‒PCR detection of osteogenic biomarker genes (*Runx2, Osterix, OCN, OPG, and RANKL*) in OBs derived from *Trim21*^*−/*−^ and *Trim21*^*+/+*^ mice upon osteogenic induction for 7 days. **g**, **h** Representative immunoblotting analysis (**g**) and quantification of Runx2 and Osterix in OBs (**h**) after osteogenic induction for 0, 4, and 7 days. **i** Representative micro-CT images of calvarial bone defects in 2-month-old *Trim21*^*+/+*^ and *Trim21*^*−/*−^ mice after surgical induction for 1 month (left panel). Quantitative measurements of bone volume per tissue volume (BV/TV) and bone defect diameter (right panel). All bar graphs are presented as the mean ± SD. *P < 0.05; **P < 0.01; ***P < 0.001; ****P < 0.000 1; n.s. not significant by Student’s *t* test

Furthermore, the expression of Trim21 was examined in MC3T3-E1 preosteoblastic cells and primary OBs after culture with osteogenic medium, and the results revealed that Trim21 protein expression gradually decreased upon osteogenic induction, accompanied by an increase in Runx2 and Osterix protein expression in a time-dependent manner (Fig. S5a–d and Fig. 2a, b). Next, we investigated the role of Trim21 in OB differentiation. As expected, OBs derived from calvarial bones of WT mice possessed a typical capacity to differentiate into osteogenic lineage cells in response to osteogenic induction (Fig. 2c), while OBs from *Trim21*^−/−^ mice exhibited an increase in Alizarin Red S and ALP staining (Fig. 2d, e) along with an elevation of *Runx2* and *Osterix* mRNA expression compared to those from the *Trim21*^+/+^ mice (Fig. 2f). Additional markers, including osteocalcin (OCN), osteoprotegerin (OPG), and receptor activator of nuclear factor kappa-B ligand (RANKL), were assessed, and the results showed that the mRNA expression of *OCN* and *OPG* was increased, accompanied by a decrease in *RANKL* mRNA expression in the pre-OBs upon the loss of Trim21 expression (Fig. 2f). Accordingly, the protein expression of Runx2 and Osterix was significantly increased in the OBs of *Trim21*^−/−^ mice upon osteogenic induction for 7 days compared with that in the OBs of *Trim21*^+/+^ control mice (Fig. 2g, h). Furthermore, the 3D reconstruction images of calvaria showed an increase in the bone regeneration area in the *Trim21*^−/−^ mice compared to the WT controls (Fig. 2i). Consistent with this finding, measurement of the BV/TV, the calvarial bone defect diameter and skeletal staining revealed a slight increase in progressive bone formation in and around the defect in the *Trim21*^−/−^mice (Fig. 2i and Fig. S5e). Taken together, these data suggest that Trim21 is crucial for OB differentiation and bone formation.

### Trim21 regulates OC differentiation and bone resorption

**Fig. 3:**
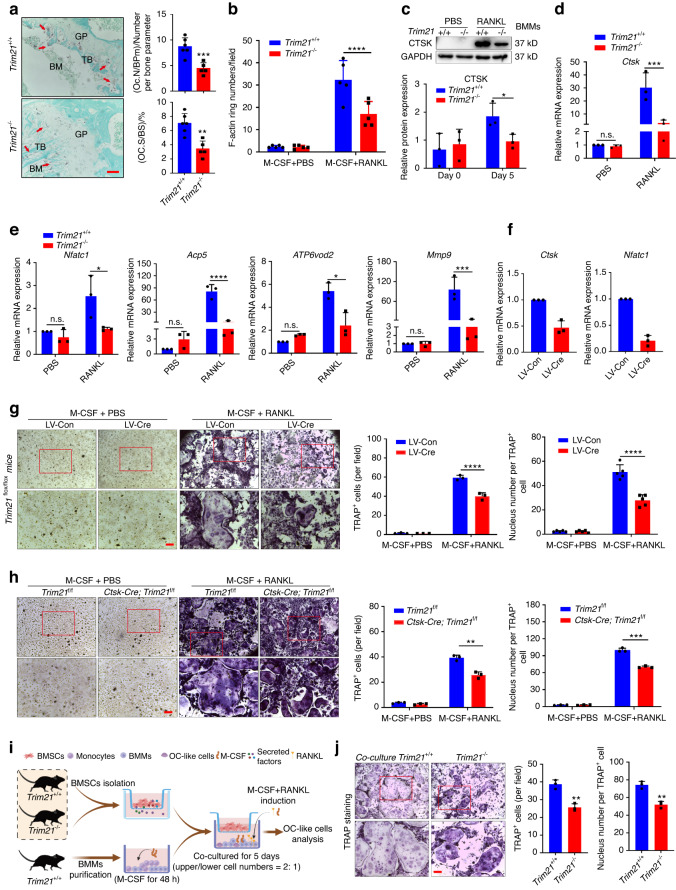
Loss of *Trim21* inhibits osteoclast formation and differentiation. **a** Representative image of histological sections of the tibia that were stained with TRAP (left panel). Bone marrow (BM) and trabecular bone (TB) are indicated in black. TRAP-stained osteoclasts (OCs) are denoted by the red arrow. OC. N/BPm (OC number per bone parameter) and OC. S/BS (OC surface per bone surface) were determined (right panel). Scale bar: 100 μm. **b** Quantification of F-actin ring number in BMM-derived OCs from immunofluorescence staining of Fig. S6e. **c** Representative immunoblot analysis and quantification of Ctsk expression in BMM-derived OCs from *Trim21*^*+/+*^ and *Trim21*^*−/−*^ mice. **d**, **e** Quantitative RT‒PCR detection of OC differentiation genes (*Ctsk, Nfatc1, Acp5, ATP6vod2, and Mmp9*) in BMM-derived OCs from *Trim21*^*+/+*^ and *Trim21*^−*/*−^ mice. PBS indicates PBS containing 30 ng·mL^−1^ M-CSF, while RANKL indicates induction with 30 ng·mL^−1^ M-CSF and 100 ng·mL^−1^ RANKL **f**, **g** Quantitative RT‒PCR detection (**f**) of OC differentiation genes (*Ctsk and Nfatc1*) and TRAP staining (**g**) in BMM-derived OCs from *Trim21*^*f/f*^ mice treated with 30 ng·mL^−1^ M-CSF plus 100 ng·mL^−1^ RANKL or PBS containing 30 ng·mL^−1^ M-CSF for 5 days and infected with lentivirus expressing EGFP or Cre recombinase (defined as LV-Con or LV-Cre, respectively). Quantification of TRAP-positive OCs and the number of nuclei per TRAP^+^ cell (right panel) (**g**). Scale bar: 50 μm. **h** BMMs derived from 4-week-old *Trim21*^*f/f*^ and *Ctsk-cre; Trim21*^*f/f*^ mice were induced for OC differentiation with either 30 ng·mL^−1^ M-CSF plus 100 ng·mL^−1^ RANKL or PBS containing 30 ng·mL^−1^ M-CSF for 5 days (left panel). Quantification of TRAP-positive OCs and the number of nuclei per TRAP^+^ cell (right panel). Scale bar: 50 μm. **i**, **j** Schematic diagram illustrating the coculture model of OCs with BMSCs from *Trim21*^*+/+*^ and *Trim21*^−*/*−^ mice (**i**). Representative images (left panel) and quantification data of TRAP-positive OCs and nucleus number per TRAP^+^ cell (right panel) (**j**). Scale bar: 50 μm. All bar graphs are presented as the mean ± SD. *P < 0.05; **P < 0.01; ***P < 0.001; ****P < 0.000 1; n.s. not significant by Student’s *t* test

Bone homeostasis is normally maintained by the tight coupling of bone formation and bone resorption. We then explored whether the higher bone mass in *Trim21*^−/−^ mice could also be driven by a decrease in OC formation and bone resorption. Of note, tartrate-resistant acid phosphatase (TRAP) staining of tibial sections from *Trim21*^−/−^ mice showed a significant decrease in TRAP-positive OCs on the TB surface compared with that in *Trim21*^+/+^ littermates (Fig. 3a). Next, we investigated the expression of Trim21 during OC differentiation into bone marrow-derived macrophages (BMMs). A suggestive increase in NFATC1 and CTSK was observed, but strong upregulation of Trim21 was observed upon OC induction (Fig. S6a, b). Consistent with this finding, BMMs derived from *Trim21*^−/−^ mice exhibited a significant inhibition of RANKL-stimulated osteoclastogenesis, as revealed by immunoblotting and TRAP staining (Fig. S6c, d). In addition, we found that BMMs from *Trim21* KO mice exhibited a specific impairment in RANKL-induced F-actin ring formation (Fig. 3b and Fig. S6e), which is known to be essential for bone resorption by activated OCs. Moreover, suppression of CTSK protein and mRNA expression was observed upon the loss of *Trim21* in BMMs cultured with OC differentiation medium (Fig. 3c, d). A series of OC markers, including *Nfact1, Acp5, ATP6vod2*, and *Mmp9*, were concomitantly suppressed in BMMs from *Trim21*^−/−^ mice compared to their control littermates, as shown by quantitative RT‒PCR (Fig. 3e). Our finding is supported by a recent study showing that *Trim21*^−/−^ BMMs had decreased expression of macrophage colony-stimulating factor (M-CSF) signature genes, which reduced their differentiation in response to a series of stimuli, including M-CSF.^23^

The above results were further verified using RAW264.7 cell lines, in which knockdown of Trim21 by the indicated siRNAs (siTrim21#2 and siTrim21#3) significantly decreased the number of TRAP^+^ cells and the mRNA levels of *Nfact1* and *Ctsk* (Fig. S6f, g). Specifically, BMMs were extracted from *Trim21*^*f/f*^ mice and then infected with lentivirus expressing EGFP or Cre recombinase (defined as LV-Con or LV-Cre, respectively), followed by the induction of OC differentiation with M-CSF and RANKL (Fig. S6h). Consistent with the results of the global knockout mice, Trim21 deficiency, induced by the introduction of Cre recombinase using LV-Cre, substantially suppressed RANKL-stimulated osteoclastogenesis in BMMs from *Trim21*^*f/f*^ mice (Fig. 3f, g). Similar results were observed in BMMs derived from *Ctsk-cre*; *Trim21*^*f/f*^ mice (Fig. 3h). Since Trim21 has been demonstrated to modulate bone formation and resorption, we next explored whether Trim21 also plays a role in regulating BMSC-mediated coupling. Notably, by the use of a coculture system, we found that BMSCs from *Trim21*^−/−^ mice remarkably impaired the formation of OCs by BMMs, in contrast to BMSCs from WT mice (Fig. 3i, j). Together, these results suggest that Trim21 positively regulates OC differentiation and maturation and that the phenotype observed in *Trim21* global and conditional knockout mice can be attributed to the suppression of bone resorption.

### YAP1/β-catenin signaling is involved in Trim21-regulated OB differentiation

**Fig. 4:**
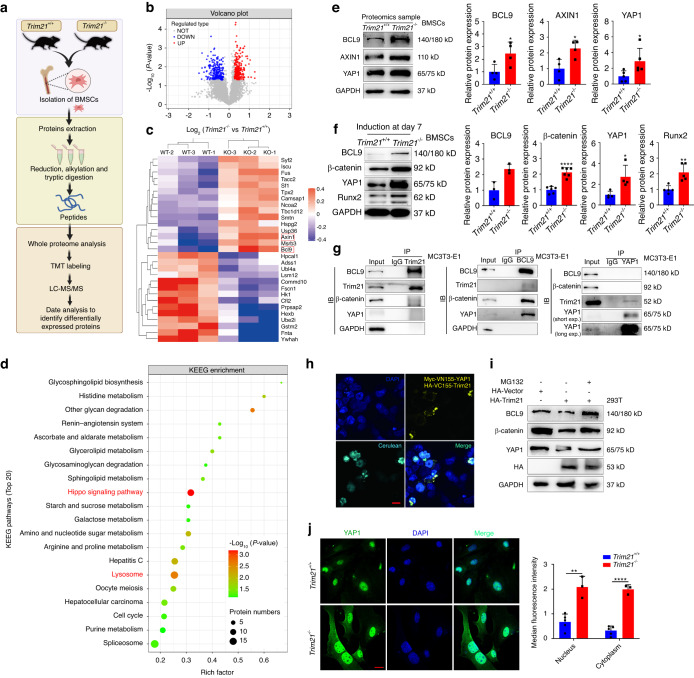
YAP1/β-catenin signaling is essential for Trim21-mediated osteogenic differentiation. **a** Schematic diagram showing TMT-based quantitative proteomics for the identification of differentially expressed proteins (DEPs) in bone marrow mesenchymal stem cells (BMSCs) derived from *Trim21*^*+/+*^ and *Trim21*^*−/−*^ mice. **b** Volcano plots of DEPs in BMSCs from *Trim21*^*+/+*^ and *Trim21*^−/−^ mice. **c** Heatmap analysis of DEPs in BMSCs. Three replicates of each group were included, and the top 29 DEPs are shown. **d** KEGG enrichment analysis of the DEPs in the BMSCs. **e** Representative immunoblotting analysis and quantification of BCL9, AXIN1, and YAP1 in BMSCs; proteomics sample: part of the samples subjected to proteomics analysis. **f** Representative immunoblotting analysis and quantification of BCL9, β-catenin, YAP1, and Runx2 protein expression in BMSCs after osteogenic induction for 7 days. **g** The endogenous interaction between Trim21, BCL9, β-catenin, and YAP1 was evaluated using a co-IP assay. **h** Protein‒protein interaction of YAP1 and Trim21 in living cells. The two BiFC plasmids encoding Myc-VN155-YAP1 and HA-VC155-Trim21 along with HA-cerulean were cotransfected into HEK293T cells for 24 h. Representative images showing transfected cells (cerulean) and the interaction between YAP1 and Trim21 (Venus). Nuclei were stained with DAPI. Scale bar: 20 μm. **i** Immunoblotting analysis of BCL9, β-catenin, YAP1, and HA-Trim21 protein expression in HEK293T cells treated with or without MG132. **j** Representative images showing the expression of YAP1^+^ OBs derived from *Trim21*^*+/+*^ and *Trim21*^*−/*−^ mice. Scale bar: 20 μm. All bar graphs are presented as the mean ± SD. *P < 0.05; **P < 0.01; ***P < 0.001; ****P < 0.000 1; n.s. not significant by Student’s *t* test

Next, we sought to identify the potential substrates or signaling pathway(s) that mediate Trim21-regulated OB differentiation using tandem mass tagging (TMT)-based quantitative proteomics (Fig. 4a). A total of 25 241 unique peptides corresponding to 4 248 proteins were identified in the mouse BMSCs derived from *Trim21*^−/−^vs. *Trim21*^+/+^ mice (Fig. S7a). Among the identified proteins, 277 and 239 proteins exhibited increased and decreased expression, respectively (Fig. S7b). Next, a volcano plot was generated to represent the protein abundance changes in BMSCs from *Trim21*^+/+^ vs. *Trim21*^−/−^ mice (Fig. 4b). To systematically identify differentially expressed proteins (DEPs) involved in OB differentiation, we generated a heatmap to visualize the hierarchical cluster analysis (Fig. 4c). Since deficiency of *Trim21* has been reported to suppress the degradation of several proteins via its E3 ubiquitin activity,^19^ we then focused on the upregulated proteins. Surprisingly, several proteins, including BCL9 and AXIN1, which are key proteins for Wnt/β-catenin signaling during OB differentiation, were found to be significantly upregulated in BMSCs from *Trim21*^−/−^ mice compared to their control cells (Fig. 4b, c). KEGG pathway analysis indicated that these DEPs are mainly enriched in the Hippo signaling pathway and lysosomes (Fig. 4d). To verify the TMT quantification results, we examined the protein expression of BCL9, AXIN1, and YAP1 (the downstream transducer of the Hippo signaling pathway) by immunoblotting analysis. As expected, compared with that in BMSCs from *Trim21*^+/+^ mice, a significant induction of BCL9, AXIN1, and YAP1 protein expression was observed in BMSCs upon Trim21 depletion in *Trim21*^−/−^ mice (Fig. 4e and Fig. S7c), indicating the reliability of our proteomic analysis.

Consistent with previous studies showing that the expression of YAP1 and β-catenin is upregulated under osteogenic induction,^24–26^ we found that loss of *Trim21* further enhanced their expression during BMSC differentiation (Fig. 4f). Interestingly, the coactivator of β-catenin, BCL9,^27^ was accordingly increased in the BMSCs and OBs from the *Trim21*^−/−^ mice (Fig. 4f and Fig. S7d). Likewise, Trim21 deficiency also increased the protein expression of β-catenin in the mouse TB (Fig. S7e). Since YAP1 has been reported to incorporate β-catenin into the destruction complex, thereby regulating bone formation,^17,25,26^ we then investigated whether there is an interaction between Trim21 and both β-catenin/BCL9 and YAP1. Notably, Trim21 was found to form a complex with BCL9/β-catenin and YAP1 upon osteogenic induction (Fig. 4g). Furthermore, a direct interaction between Trim21 and YAP1 was examined using a bimolecular fluorescence complementation (BiFC) assay, which is a valuable tool for studying protein‒protein interactions in their native cellular environment.^28^ Notably, the direct interaction was demonstrated by reconstituted Venus fluorescence (BiFC signal) after the transfection of Myc-VN155-YAP1 with HA-VC155-Trim21 into HEK293T cells (Fig. 4h). Next, we sought to determine how Trim21 regulates YAP1 expression. Overexpression of Trim21 (HA-Trim21) significantly suppressed YAP1 expression, while treatment with MG132 attenuated the inhibitory effect of HA-Trim21 on YAP1 expression (Fig. 4i and Fig. S7f). A similar degradation pattern was observed for β-catenin and BCL9 (Fig. 4i and Fig. S7f), indicating that the stability of the BCL9/β-catenin/YAP1 protein complex is likely to be regulated by Trim21 through a proteasomal degradation pathway. In support of this, the loss of Trim21 stabilized cytoplasmic YAP1 expression and favored its translocation into the nucleus, and the depletion of YAP1 by verteporfin (VP) led to the inactivation of BCL9/β-catenin/TCF-1 signaling during osteoblast differentiation (Fig. 4j and Fig. S7g,h). Collectively, our data indicate that loss of *Trim21* abolishes the cytoplasmic degradation of the YAP1/BCL9/β-catenin complex, which enables the activation of OB differentiation via YAP1/β-catenin-dependent signaling.

### Depletion of *Trim21* prevents pathological bone loss by activating YAP1 signaling

**Fig. 5:**
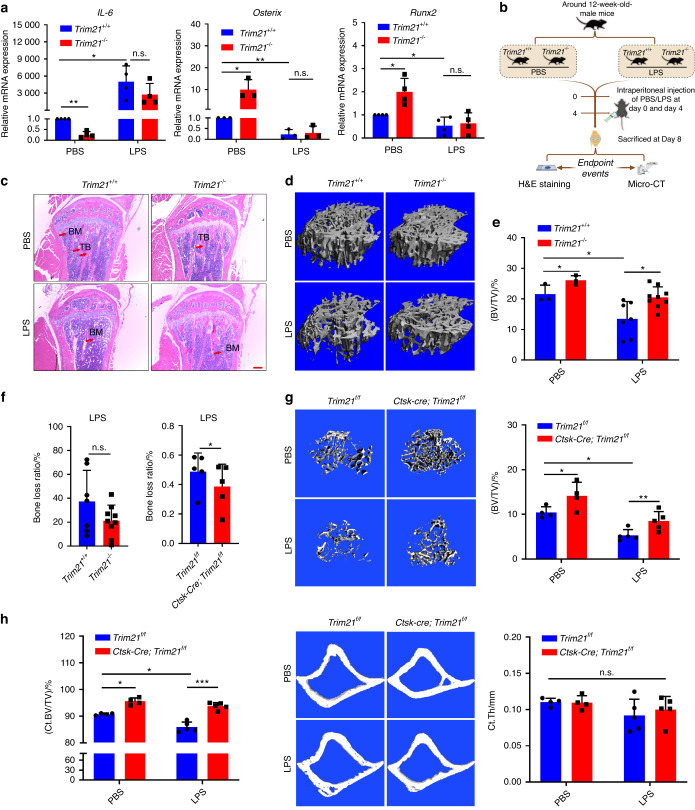
Loss of *Trim21* protects mice from lipopolysaccharide (LPS)-induced bone loss. **a** Quantitative RT‒PCR determination of *IL-6*, *Osterix*, and *Runx2* mRNA expression in OBs with or without lipopolysaccharide (LPS) treatment during osteogenic induction. **b** Schematic diagram showing the H&E staining and micro-CT analysis of *Trim21*^*+/+*^ and *Trim21*^−*/−*^ mice induced by PBS or LPS. **c** Representative images of H&E staining of tibia sections of 13-week-old *Trim21*^*+/+*^ and *Trim21*^−/−^ mice induced by PBS or LPS. Bone marrow (BM) and trabecular bone (TB) are labeled with red arrows. **d**, **e** Representative micro-CT images (**d**) and BV/TV (**e**) of proximal tibia trabecular bone of 13-week-old *Trim21*^*+/+*^ and *Trim21*^−/−^ mice induced by PBS or LPS. **f** The bone loss ratio after LPS treatment in global knockout mice (left panel) and conditional knockout mice (right panel)**. g** Representative micro-CT images and BV/TV of proximal tibia trabecular bone of 13-week-old *Trim21*^*f/f*^ and *Ctsk-cre; Trim21*^*f/f*^ mice induced by PBS or LPS. **h** Representative micro-CT images of cortical bones and quantification of BV/TV (left panel) and thickness (right panel) of *Trim21*^*f/f*^ and *Ctsk-cre; Trim21*^*f/f*^ mice induced by either PBS or LPS. All bar graphs are presented as the mean ± SD. *P < 0.05; **P < 0.01; ***P < 0.001; n.s. not significant by Student’s *t* test

Given the fundamental role of Trim21 in immunity,^23,29,30^ we also examined the role of Trim21 in inflammation-induced bone loss both in vitro and in vivo. Treatment with TNF-α or LPS significantly stimulated the expression of IL-6 and Trim21, accompanied by a reduction in Osterix and Runx2 protein expression in MC3T3-E1 cells (Fig. S8a–c). Similarly, treatment with LPS in OBs from WT mice resulted in a suppression of *Runx2* and *Osterix* mRNA expression and an induction of *IL-6* mRNA levels; however, these effects were significantly attenuated in OBs from *Trim21*^−/−^ mice (Fig. 5a), indicating that Trim21 may have a protective effect against the impact of LPS on OBs. Furthermore, LPS injection was found to cause a significant decrease in bone mass in the proximal tibia of WT mice, yet less bone loss, as determined by micro-CT, was observed in *Trim21* KO mice upon LPS administration (Fig. 5b–f and Fig. S8d). Furthermore, *Ctsk-cre; Trim21*^*f/f*^ mice were used to confirm the above findings. As expected, conditional knockout of *Trim21* partially protected against bone loss induced by LPS, as evidenced by a significant increase in bone volume per tissue volume (BV/TV), trabecular thickness (Tb. Th), and trabecular number (Tb. N) and a decrease in trabecular separation (Tb. Sp) (Fig. 5f, g and Fig. S8e). Similar to the changes in TB, the BV/TV of cortical bone in *Ctsk-cre*; *Trim21*^*f/f*^ mice was found to be slightly increased compared to that in their *Trim21*^*f/f*^ littermates, although cortical bone thickness did not change significantly (Fig. 5h). These results together demonstrate that Trim21 plays a crucial role in regulating bone mass, particularly in the context of inflammation.

**Fig. 6:**
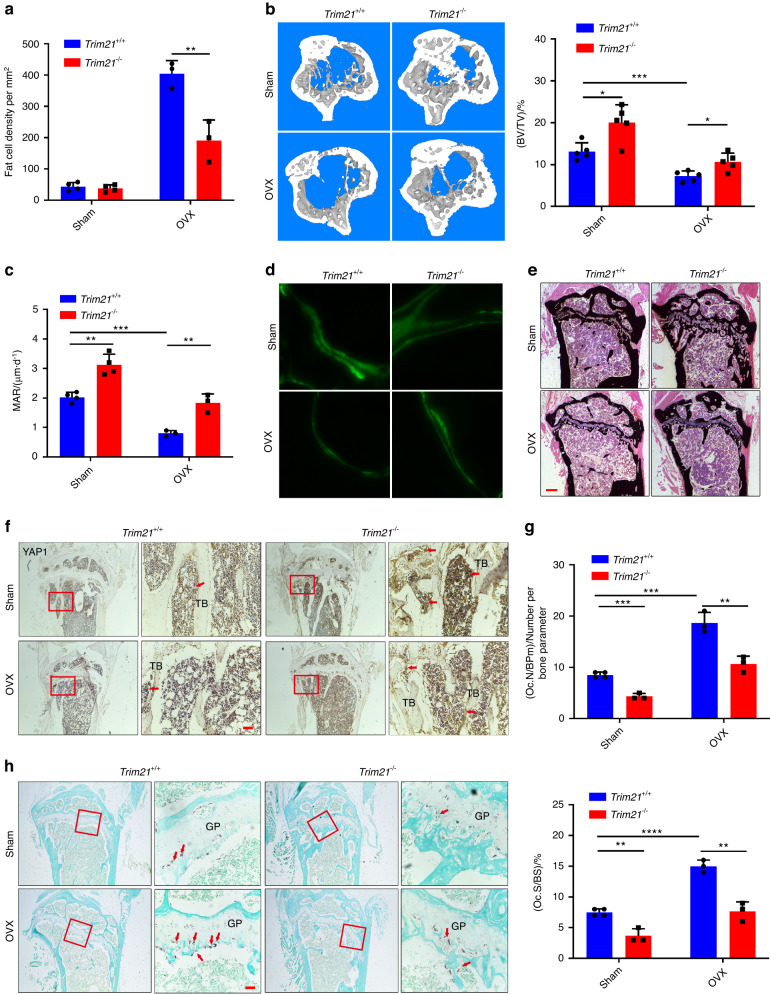
Trim21 orchestrates ovariectomy (OVX)-induced bone metabolism by targeting YAP1 signaling. **a** Determination of fat cell density in the proximal tibia of 20-week-old *Trim21*^*+/+*^ and *Trim21*^−/−^ mice induced by sham operation or OVX. **b** Representative micro-CT images and quantitative data (BV/TV) of proximal tibial bone of 20-week-old *Trim21*^*+/+*^ and *Trim21*^−/−^ mice induced by sham operation or OVX. **c**, **d** Calcein double labeling of mineral layers of tibial trabecular bone of 5-month-old mice. **e** Representative images of von Kossa staining of the undecalcified proximal tibia of 5-month-old mice. Scale bar: 50 μm. **f** IHC staining images of the proximal tibia of 5-month-old mice using an antibody against YAP1. The YAP1-stained positive cells are denoted by the red arrow. Scale bar: 50 μm. **g**, **h** Representative images of histological sections of the tibia that were stained with TRAP in *Trim21*^+/+^ and *Trim21*^−/−^ mice induced by sham operation or OVX. TRAP-stained osteoclasts (OCs) are denoted by the red arrow. OC. N/BPm (OC number per bone parameter) and OC. S/BS (OC surface per bone surface) was determined. Scale bar: 100 μm. All bar graphs are presented as the mean ± SD. *P < 0.05; **P < 0.01; ***P < 0.001; ****P < 0.000 1; n.s. not significant by Student’s *t* test

For further elucidation of the role of Trim21 in osteoanabolic function in pathological bone loss, *Trim21*^+/+^ and *Trim21*^−/−^ mice were sham-operated or underwent an OVX at 12 weeks of age to mimic estrogen deficiency-induced osteoporosis, followed by histological and micro-CT analysis 8 weeks later (Fig. S9a). Estrogen deficiency-induced osteoporosis exhibits a typical phenotype characterized by low bone mass and excessive accumulation of adipose tissue in the bone marrow milieu;^3,9^ consistently, *Trim21*^+/+^ OVX-induced mice displayed thinned osteoporotic trabeculae with lost continuity, enlarged areolae, and increased fat cell density, whereas *Trim21-*deficient mice showed partial reversal of the OVX-induced phenotype (Fig. 6a and Fig. S9b). These findings were further confirmed by micro-CT analysis, which showed a slight suppression of bone loss induced by OVX (Fig. 6b and Fig. S9c).

**Fig. 7:**
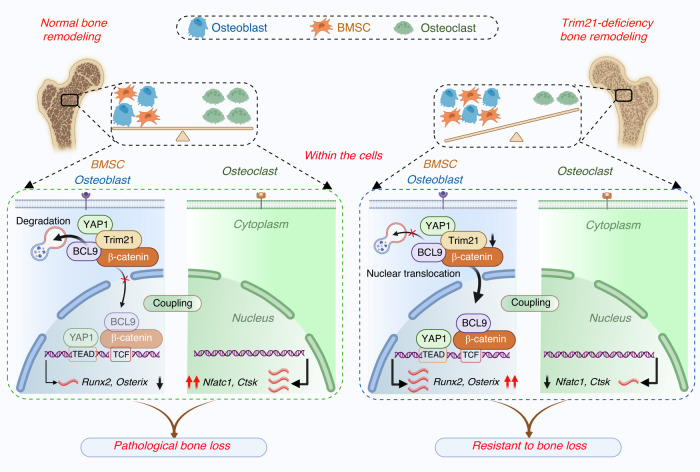
A schematic of *Trim21* in the regulation of bone remodeling via YAP1/β-catenin signaling. Normal bone remodeling is maintained by the balance of MSC/osteoblast-mediated bone formation and osteoclast-mediated bone resorption. Trim21, by interacting with the protein complex formed by YAP1/β-catenin/BCL9, dictates the degradation of this protein complex, which in turn inactivates YAP1 and β-catenin signaling, which is essential for osteoblast differentiation. However, Trim21 is critical for maintaining the basic expression of osteoclast biomarkers, including *Nfatc1* and *Ctsk*. Therefore, the coupling of osteoblasts with osteoclasts is attributed to dynamic changes in bone metabolism (left panel). In contrast, the loss of *Trim21* causes disassociation with the YAP1/β-catenin/BCL9 complex, which then enters the nucleus for subsequent activation of osteogenic genes, including *Runx2 and Osterix*. In addition, the loss of *Trim21* suppresses the maturation of osteoclasts. Together, these results indicate that *Trim21* deficiency alleviates pathological bone loss by activating YAP1/β-catenin signaling

To further assess the role of Trim21 in bone formation, we performed dynamic bone histomorphometric analysis of the proximal tibia using double-calcein labeling, von Kossa staining, IF/IHC, and TRAP staining in the OVX-induced bone loss model (Fig. S9d). The results showed that the mineral apposition rate (MAR) was significantly higher in *Trim21*^−/−^ *mice* than in control littermates with or without OVX induction (Fig. 6c, d). The results of von Kossa staining of undecalcified tibia sections further confirmed the enhanced calcium deposition in *Trim21*^−/−^ mice (Fig. 6e), indicating the enhancement of bone mineralization and OB maturation. Accordingly, there was a significant increase in the presence of YAP1-stained OBs around the TB in *Trim21*^−/−^ mice compared to mice with intact Trim21 (Fig. 6f). In contrast, Trim21 deficiency significantly decreased the presence of OCs (TRAP-positive cells) on the surface of TBs induced by OVX (Fig. 6g, h). Collectively, these results highlight the promising role of Trim21 in dual-targeting of OBs and OCs for the treatment of skeletal degenerative disorders, including osteoporosis (Fig. 7).

## Discussion

Recently, studies aiming to develop dual-action treatments for suppressing bone resorption while promoting bone formation have emerged and are promising in the field of osteoporosis. For instance, romosozumab, a dual-action drug that enhances bone formation and simultaneously suppresses bone resorption, has been approved by the U.S. FDA as a first-in-class sclerostin inhibitor.^31^ In addition, a recent study using conditional knockout of sialic acid-binding immunoglobulin-like lectin-15 (Siglec-15) demonstrated that a neutralizing antibody against Siglec-15 caused a decrease in mature multinuclear OCs and an increase in bone formation via the promotion of PDGF-BB production.^32^ This dual-action target shows significant translational potential as a novel therapy for osteoporosis and bone fracture. In the present study, we demonstrated that Trim21 is crucial for regulating OB-mediated bone formation and OC-mediated bone resorption, thereby providing a basis for exploring *Trim21* as a novel dual-targeting approach for treating pathological skeletal diseases, including osteoporosis.

Recently, a subset of TRIM family members was shown to orchestrate bone homeostasis. For instance, TRIM38 plays an essential role in bone remodeling by facilitating OB differentiation and suppressing OC differentiation via negative regulation of NF-κB activity.^13^ In addition, Trim16, Trim33, and Trim63 have been shown to promote OB differentiation. In contrast, Trim14 and Trim44 have been found to promote OC differentiation.^15,33,34^ Herein, we reveal that Trim21 is a negative regulator of bone metabolism. First, knockout of *Trim21* elevated bone mass and protected mice from OVX- and LPS-induced bone loss in vivo (Fig. 1 and Fig. 5). Second, *Trim21* deficiency promoted bone formation and osteogenic differentiation by increasing the expression levels of osteogenic genes, including Runx2 and Osterix, in mouse BMSCs, calvarial OBs, and preosteoblastic MC3T3-E1 cell lines (Fig. 2, Fig. 6, and Figs. S3–4). This finding is consistent with previous studies by our group and others showing that Trim21 impeded OB differentiation of osteosarcoma or MSCs by inducing the degradation of Runx2 or Akt, respectively.^19,20^ A recent study demonstrating that Trim21 negatively regulates Runx2 expression via the interaction with the SET domain containing lysine methyltransferase 7/9 (SET7/9) also supports our conclusion.^35^ Third, loss of *Trim21* significantly suppressed RANKL-induced OC differentiation in primary BMMs and RAW264.7 cells and reduced the number of TRAP-positive multinucleated cells on the TB surface induced by OVX (Fig. 3, Fig. 6, and Fig. S5).

Next, we explored the underlying regulatory mechanisms of Trim21 in bone remodeling and OB differentiation in particular. Trim21 is an E3 ubiquitin ligase that prepares proteins for proteasomal and lysosomal degradation.^36^ Our recent work demonstrated that the Trim21/ANXA2/TFEB axis is critical for the regulation of OB differentiation via autophagy in osteosarcoma.^19^ In the present study, unbiased screening of *Trim21-*deficient mouse BMSCs revealed that YAP1, by cooperation with BCL9/β-catenin signaling, is crucial for Trim21-regulated bone remodeling (Fig. 4 and Fig. 6). Previous studies have demonstrated that YAP1 is a key transcriptional coactivator of the Hippo pathway for promoting bone formation.^24^ Moreover, YAP1 has been shown to crosstalk with Wnt/β-catenin and thus control bone mass and bone homeostasis.^37^ In other studies, YAP1 was reported to function upstream of β-catenin signaling, and its role in regulating osteoblast differentiation and bone formation was attributed to β-catenin signaling.^24,38,39^ Herein, it is more likely that β-catenin is a downstream molecule of YAP1 that is required for Trim21-regulated bone remodeling, given that inhibition of YAP1 by VPs strongly diminished the OB differentiation of BMSCs from *Trim21*^−/−^ mice (Fig. S7). In addition, given that one of the key functions of TCF/LEF proteins is to facilitate the localization of β-catenin within the cells and regulate the transcription of Wnt target genes,^40^ our results support the involvement of TCF/LEF proteins in Trim21-regulated osteoblastic and adipogenic differentiation and bone homeostasis (Figs. 2, 4, S4, and S8). The use of conditional knockout mice will help to further clarify the crosstalk between inflammation and YAP1/β-catenin signaling in the context of osteoporosis.

Previous studies have revealed that an increase in NF-κB-p65 phosphorylation promotes the secretion of a series of inflammatory cytokines in OVX-induced mice or postmenopausal osteoporosis patients.^41,42^ Given the fundamental role of Trim21 in regulating immunity via ubiquitination of the nuclear factor‐κB p65 subunit,^30,43^ it would be interesting to further determine whether the regulation of Trim21 in bone metabolism can partially be attributed to the mediation of immunity. Moreover, another limitation of the study is that tibial bone specimens were used to assess the expression of Trim21, which was then correlated with OP status due to the difficulty in obtaining relatively normal femoral bone during total hip arthroplasty. Thus, further investigation is warranted to comprehensively examine the impact of copathologies associated with OA on OP to provide a more holistic understanding of these conditions.

In conclusion, our results found that Trim21 is elevated in osteoporosis patients and that its deficiency leads to high bone mass compared with that of control littermates in sham-operated and OVX-induced mice. We further demonstrated that *Trim21* depletion favors bone formation by enhancing the osteogenic differentiation of BMSCs and elevating the activity of OBs, whereas loss of *Trim21* suppressed OC formation and maturation. Mechanistically, YAP1/β-catenin signaling was identified and demonstrated to be activated upon the loss of *Trim21* in BMSCs and in vivo bone specimens. Our study highlights the potential therapeutic use of Trim21 as a promising dual-targeting candidate for preventing bone loss and reducing fracture risk in osteoporosis patients.

## Materials and methods

### Human bone tissue collection

All experiments were approved by the Ethics Committee of the First Affiliated Hospital of Jinan University (Ethical approval number: KY-2021-065). All patients aged 49–81 years old were subjected to an examination of serum bone metabolic biomarkers: osteocalcin (OST), procollagen type I N-terminal propeptide (PINP), C-terminal cross-linking telopeptide of type I collagen (β-CTx), and alkaline phosphatase (ALP). Bone mineral density (BMD) was assessed using dual-energy X-ray absorptiometry (DXA) and was categorized into three groups: normal (femoral neck (FN)-BMD T score > −1.0), osteopenia ( − 2.5 < FN-BMD T score ≤ −1.0), and osteoporosis (FN-BMD T score ≤−2.5). Participants with other metabolic diseases and abnormalities in any of the screening laboratory tests (complete blood count, serum calcium, phosphorus, albumin, etc.) were excluded from our study. The detailed characteristics of each patient are listed in Table S1. Human tibial bone specimens were obtained from patients who received total knee arthroplasty at the Department of Bone and Joint Surgery of the First Affiliated Hospital of Jinan University and were immediately placed in a liquid nitrogen jar for preservation. All participants were aware of the purpose of the study and signed informed consent before participating in the study.

### Establishment of global and conditional *Trim21* knockout mice

Heterozygous recombinant Trim21 mice (*Trim21*^−/+^) with a C57BL/6 genetic background were purchased from Cyagen Biosciences (Guangzhou, China). Heterozygous recombinant Trim21 mice (*Trim21*^flox/+^) with a C57BL/6 genetic background were purchased from GemPharmatech (Nanjing, China). Animals were bred and maintained under specific pathogen-free (SPF) conditions. All animal procedures were approved by the Institutional Animal Care and Use Committee of Jinan University (Approval number: IACUC-20211216-15 and IACUC-202111213-02, respectively) and conformed to the “Guide for the Care and Use of Laboratory Animals” of the National Institute of Health in China. Briefly, *Trim21* knockout (KO) (*Trim21*^−/−^) mice and WT (*Trim2*^+/+^) mice were generated by intercrossing the heterozygous targeted mice, followed by genotyping with a Quick Genotyping Assay Kit for Mouse Tail (Beyotime, Cat# D7283S, China). For the generation of Trim21 conditional knockout mice (*Ctsk-cre; Trim21*^*f/f*^) in OCs, *Trim21*^*f/f*^ mice were crossed with *Ctsk-cre* mice. All mice were on a C57BL/6 background. Polymerase chain reaction (PCR) genotyping primers are listed in Table S2.

### Cell culture and OB treatments

Bone marrow mesenchymal stem cells (BMSCs) were derived from 4- to 6-week-old *Trim21*^+/+^ and *Trim21*^−/−^ mice. Primary murine calvarial OBs were isolated from 3-day-old mice. The preosteoblast cell line MC3T3-E1 was gifted by Prof. Chengzuo Qiu (Jinan University, Guangzhou). HEK293T cells were purchased from the American Type Culture Collection (ATCC, USA) and were maintained in our laboratory and regularly tested for mycoplasma contamination.^44^ BMSCs, OBs, and MC3T3-E1 cells were cultured in complete α-MEM containing 10% FBS and 1% penicillin/streptomycin.

For protein degradation evaluation, HEK293T cells were seeded into (2 × 10^5^ cells per well) 6-well plates for 24 h. Cells were then transiently transfected with 1 μg of plasmid encoding HA-Vector or HA-Trim21 using Lipofectamine 2000 (Thermo Fisher Scientific, Cat# 11668019, USA) for the indicated time, followed by treatment with 5 μmol·L^−1^ MG132 (Selleck, Cat# S2619, USA) for another 24 h and immunoblotting analysis.

For osteogenic differentiation, BMSCs and OBs were reseeded at a density of 5 × 10^5^ cells per well into 6-well plates or 2.5 × 10^5^ cells into 12-well plates. BMSCs were then stimulated with a MesenCult Osteogenic Stimulatory Kit (Mouse) (StemCell, Cat# 0504, StemCell, Canada). MC3T3-E1 cells were seeded at a density of 2 × 10^5^ cells per well into 6-well plates or 1 × 10^5^ cells into 12-well plates. OBs and MC3T3-E1 cells were stimulated with osteogenic medium containing 10% FBS, 50 μg·mL^−1^ ascorbate, 10 μmol·L^−1^ β-glycerophosphate, 0.1 μmol·L^−1^ dexamethasone, and 10 μmol·L^−1^ glutamine. The osteogenic medium was replaced every 3 days for all cells.

To mimic inflammation-induced bone loss in vitro, we seeded MC3T3-E1 cells into 6-well plates at a density of 2.5 × 10^5^ for 24 h. Then, the cells were treated with different concentrations of LPS (Beyotime, Cat# S1732, China) or TNF-α (CST, Cat# 89025 C, USA) for another 24 h, followed by the analysis of Trim21, IL-6, and osteogenic markers (Runx2 and Osterix) by quantitative RT‒PCR or immunoblotting assays.

### Osteoclastogenesis in BMMs and the coculture system

BMMs were derived from 4- to 8-week-old *Trim21*^+/+^ and *Trim21*^−/−^ mice or *Trim21*^*f/f*^ mice, as well as *Ctsk-cre; Trim21*^*f/f*^ mice, and then incubated in α-MEM containing 30 ng·mL^−1^ M-CSF (MCE, Cat# HY-P70553, USA) for 4 days to generate BMMs. For generation of mature OCs, BMMs were cultured with 30 ng·mL^−1^ M-CSF and 100 ng·mL^−1^ RANKL for at least 5 days, followed by a series of experiments. The same volume of phosphate-buffered saline (PBS) containing 30 ng·mL^−1^ M-CSF was applied as a control. For specific knockout of *Trim21*, BMMs from *Trim21*^*f/f*^ mice were infected with lentivirus expressing EGFP or Cre recombinase (defined as LV-Con or LV-Cre, Obio Technology, China).

RAW264.7 monocytes/macrophages (ATCC, USA) were cultured and maintained as we described previously.^45^ For induction of the differentiation of RAW264.7 cells into OCs, 100 ng·mL^−1^ RANKL (R&D Systems, Cat# 462-TEC-010) was added to the cells for three more days, and the medium was changed every 2 days.

In the coculture model of BMSCs-BMMs, BMSCs from *Trim21*^+/+^ and *Trim21*^−/−^ mice were placed in the upper chamber of Transwell inserts (Costar, Cat#3450, USA), while BMMs from *Trim21*^+/+^ mice were seeded in the lower chamber at a density of 4 × 10^5^ cells per well with 30 ng·mL^−1^ M-CSF and 100 ng·mL^−1^ RANKL induced for 5 days, allowing cell‒cell communication without direct physical contact. This Transwell configuration allows a specific ratio of 2:1 for BMSCs to BMMs.

### Plasmid construction

The pCMV-FLAG vector was gifted by Prof. Chang-Deng Hu (Purdue University). For cloning of mouse Trim21 genes, the cDNA of RAW264.7 cells was amplified by PCR using the primers 5′-CCGAATTCTCATGTCTCTGGAAAAGATGTGGG-3′ and 5′- TAAAGCGGCCGCTCACATCTTTAGTGGACAGAGCTT-3′. The purified PCR fragment was cloned and inserted into the abovementioned backbone using EcoRI and NotI sites for the construction of pCMV-FLAG-mus-Trim21. For construction of Myc-VN155-YAP1, the YAP1 gene was amplified from HA-YAP1 using the primers 5′-GCAGATCTGGATGGATCCCGGGCAGCAGCCG-3′ and 5′-TAAAGCGGCCGCCTATAACCATGTAAGAAAGCTTTCT-3′ and then subcloned and inserted into the BglII-NotI sites of pBiFC-VN155.^44,46^ The above constructs were verified by sequencing and immunoblotting analysis. The pCMV-HA-homo-Trim21 was previously constructed.^47^

### Small interfering RNA assays

siRNA targeting Trim21 was purchased from GenePharma (Shanghai, China). The sequences are listed in Table S3. Transfection of siRNA was performed using Lipofectamine 2000 (Thermo Fisher, Cat# 11668019, USA) according to our previous study.^47^ Briefly, MC3T3-E1 or RAW264.7 cell lines were seeded in 6-well plates at a density of 2 × 10^5^ cells per well for 24 h and then transiently transfected with 5 μL of Lipofectamine 2000 and 5 μL of siCon, *siTrim21#2* or *siTrim21#3* for 4 h. Then, the medium was changed to complete α-MEM, and the knockdown effect of *Trim21* was verified by quantitative RT‒PCR and immunoblotting after 48 h.

### Alizarin red S, ALP, and oil red O staining

Cells were incubated with osteogenic medium for 7–14 days to allow the formation of opaque calcified nodules. The cell samples were then washed once with phosphate-buffered saline (PBS), followed by fixation with 4% paraformaldehyde (PFA) for 20 min. Washed nodules were then stained with 0.1% Alizarin Red S (Beyotime, Cat# C0148S, China) solution for 30 min. Alkaline phosphatase (ALP) staining was carried out using an ALP staining kit (KeyGen, Cat# KGA353, China). After 1 week of adipogenic induction in BMSCs, the cells were subjected to Oil Red O staining according to the manufacturer’s protocols (Beyotime, Cat# C0158S, China). The stained images were observed and taken using a phase-contrast microscope (Nikon, Tokyo, Japan). The area stained by Alizarin Red S, ALP, or Oil Red O was quantified using ImageJ software (National Institutes of Health, Bethesda, MD, USA) from over 5 random fields.

### Tartrate-resistant acid phosphatase (TRAP) staining

Cells and mouse knee sections were fixed and stained with TRAP solution using a kit according to the manufacturer’s instructions (Sigma-Aldrich, Cat# 387 A, Germany). Cells with more than 3 nuclei were considered OCs. Five high-power fields (200 ×) were randomly selected for OC counting. Mouse knee sections were selected at 4 high-power fields (200 ×) in the tibial metaphysis for OC counts. TRAP-positive cells were visualized, and the number of OCs per field and the number of nuclei per TRAP^+^ cell were quantified by ImageJ software (National Institutes of Health, Bethesda, MD, USA).

### Quantitative real-time PCR (RT‒PCR)

For mRNA expression analysis, total RNA was purified using TRIzol (TaKaRa, Cat# 9109, Japan) and was reverse-transcribed into cDNA using the High-Capacity cDNA Reverse Transcription Kit (Thermo Fisher, Cat# 4368814, USA). In brief, the cancellous bone specimens were fragmented, and TRIzol was added to lyse cells to purify mRNA, followed by the reverse transcription of cDNA and quantitative RT‒PCR. Quantitative RT‒PCR was performed on a QuantStudio 5 using Fast SYBR Green PCR Master Mix (Thermo Fisher, Cat# 4385612, USA) with 500 nmol·L^−1^ primers. The primers used for quantitative RT‒PCR are listed in Table S4. The relative expression levels of the indicated genes were calculated using the 2^−ΔΔCt^ method, with expression levels normalized to that of GAPDH.^46^

### Coimmunoprecipitation (Co-IP)

Co-IP assays were performed to explore the endogenous interaction among BCL9, YAP1, and Trim21 in MC3T3-E1 cells following the same procedure as described previously.^19,47^ Briefly, cells with or without induction were harvested and then lysed by sonication in Western and IP Lysis Buffer (Beyotime, Cat# P0013, China) containing NaF, PMSF, Na_3_VO_4_, and protease inhibitors. After incubation on ice for 30 min, 40 μL of 50% protein A agarose bead slurry (CST, Cat# 9863, USA) was used to preclear cell extracts for 30 min at 4 °C, followed by incubation with antibodies against YAP1 (CST, Cat# 14074, USA, 1:50), BCL9 (Abcam, Cat# ab37305, UK, 1:50), Trim21 (Novus, Cat# NBP1-33548, China, 1:100) or control IgG (CST, Cat# 2729, USA) overnight at 4 °C. Antibody complexes were then incubated with 40 μL of 50% protein A agarose bead slurry for 4 h at 4 °C on a rotating platform. Immunoprecipitated proteins were washed five times with cold lysis buffer and then subjected to immunoblotting analysis. HRP AffiniPure Mouse Anti-Rabbit IgG Light Chain (Abbkine, Cat# A25022, USA) was used as the secondary HRP-conjugated antibody to avoid interference from the IgG heavy chain.

### Immunoblotting assay

Protein concentration was determined by the BCA method, and 20–50 µg of protein was first separated by 8%–15% sodium dodecyl sulfate‒polyacrylamide gel electrophoresis (SDS‒PAGE) gels (Beyotime, Cat# P0012A, China), followed by visualization with primary and secondary antibodies using a Tanon 5200 Luminescent Imaging Workstation (Tanon, China) as described previously^47^. Briefly, the lumbar spine of the mouse was dissected by removing the surrounding muscles and the transverse and spinous processes with a rongeur. Then, the cone and lamina of the lumbar vertebra were disrupted using a tissue disrupter with the aid of liquid nitrogen, followed by the addition of Western and IP Lysis Buffer (Beyotime Biotechnology, P0013). The protein supernatant was collected after centrifugation for subsequent experiments. Samples for other cell experiments were prepared routinely. The antibodies used were as follows: YAP1 (CST, Cat# 14074, USA, 1:1 000), β-catenin (CST, Cat# 8480 S, USA, 1:1 000), GAPDH (CST, Cat# 2118, USA, 1:1 000), FLAG (CST, Cat# 8146, USA, 1:1 000), Trim21 (Novus, Cat# NBP1-33548, China, 1:1 000), AXIN1 (CST, Cat# 2087 S, USA, 1:1 000), Osterix (Abcam, Cat# ab209484, UK, 1:1 000), BCL9 (Abcam, Cat# ab37305, UK, 1:1 000), CTSK (Abcam, Cat# ab19027, UK, 1:1 000), NFATC1 (SANTA, Cat# sc-7294, USA, 1:1 000), and Runx2 (Abcam, Cat# ab236639, UK, 1:1 000).

### Immunofluorescence (IF) staining of cells

OCs or OBs induced as described above were then fixed with 4% PFA for 20 min, permeabilized with 0.1% (v/v) Triton X-100 for 10 min, and blocked with 5% skim milk for 1 h. Cells were then incubated with primary antibodies (YAP1, CST, Cat# 14074, USA, 1:50) at 4 °C overnight, washed with phosphate-buffered saline with Tween 20 (PBST) 3 times for 5 min, and then incubated with a fluorescent secondary anti-mouse antibody (Alexa Fluor 488, green, CST, Cat# 8878, USA, 1:100). F-actin (only for OCs) was stained with rhodamine phalloidin (Invitrogen, Cat# R415, USA) in the dark for 1 h followed by 4′, 6-diamidino-2-phenylindole (DAPI) staining for 10 min. The images were captured using a laser scan confocal microscope (Zeiss LSM 880, Germany). F-actin rings were visualized, and the number of F-actin rings per field (4 ×) was quantified by ImageJ software.

### Biomolecular fluorescence complementation (BiFC) assay

HA-VC155-Trim21 was previously constructed, and the BiFC assay was performed essentially as previously described to analyze the interaction between YAP1 and Trim21 in living cells.^47^ In brief, HEK293T cells were seeded on coverslips in a 15 mm confocal dish for 24 h, and the plasmids encoding Myc-VN155-YAP1 and HA-VC155-Trim21, along with HA-cerulean, were cotransfected into cells for 4 h by Lipofectamine 2000 (Thermo Fisher, Cat# 11668019, USA). After 48 h of culture, the cells were fixed with 4% paraformaldehyde and then stained with DAPI for 5 min, followed by observation using a confocal microscope.

### Tandem mass tag-based quantitative proteomics

Tandem mass tag (TMT)-based quantitative proteomic analysis was performed by Shanghai Applied Protein Technology Company (Shanghai, China). In brief, proteins were extracted using SDT lysis buffer (4% sodium dodecyl sulfate (SDS), 100 mmol·L^−1^ Tris/HCl pH 7.6, 0.1 mol·L^−1^ dithiothreitol (DTT)), and the protein content was determined using the BCA method. Next, protein samples were digested using the filter-aided proteome preparation (FASP) method as previously described^48^ and were desalted on C18 cartridges (Empore™ SPE Cartridges C18, standard density), dried under a vacuum, and then resuspended in 0.1% (v/v) formic acid. Each set of eluted peptides was labeled with a unique TMT isobaric tag (TMT126–128 for *Trim21*^+/+^, TMT129-131 for *Trim21*^−/−^) and analyzed by a Q Exactive mass spectrometer (MS, Thermo Fisher Scientific) coupled with an Easy-nLC 1000 system (Thermo Fisher Scientific). MS raw data were analyzed by Proteome Discoverer (Thermo Fisher Scientific, version 1.4) and then subjected to a database search using the MASCOT search engine (Matrix Science, Boston, MA, USA, version 2.2) for peptide identification. In this study, a 1.2-fold change (upregulation or downregulation) was used as a cutoff for biological significance based on the standard deviation and normalized peptide ratios. The top BLAST hits were represented by volcano plots and hierarchical clustering analysis. Pathway analysis was performed using the Kyoto Encyclopedia of Genes and Genomes database (http://www.genome.jp/kegg/pathway.html).

### Double staining of the mouse skeleton

Mice were eviscerated, the skin was removed, and the resulting samples were transferred into acetone for 48 h after overnight fixation in 95% ethanol. Skeletons were then stained in Alcian blue and Alizarin Red S solution (Beyotime, Cat# C0148S, China) for 3 days at 37 °C and sequentially cleared in 1% KOH. Skeletons were then replaced in 1% KOH/20% glycerol for 3 days and passed through increasing concentrations of 1:1 glycerol/ethanol solution (20%, 50%, and 100%) for 1 day. Finally, the stained skeletons were dehydrated in glycerol for imaging and storage.

### Mouse cranial bone defect model

Twelve-week-old *Trim21*^+/+^ or *Trim21*^−/−^ mice (*n* = 5) were anesthetized by intraperitoneal injection of pentobarbital sodium (80 mg·kg^−1^), and then, the region of interest was disinfected twice. A 1.5 cm skin incision was made longitudinally along the midline of the skull to expose the parietal bone. After separation of the periosteum, a 2.5 mm hole was created in the left parietal bone far from the cranial suture using a Mini electric drill with a 2.5 mm diameter diamond bit (Shenzhen, China) at a low speed ( < 1 500 r·min^−1^). Next, the operation area was washed with PBS and then sutured. Four weeks after defect creation, mice were euthanized, and the cranium of the mice was fixed with 4% PFA solution for 48 h. Finally, the samples were transferred to 70% ethanol until processing for micro-CT and histology.

### Ovariectomy (OVX)- and lipopolysaccharide (LPS)-induced osteoporosis mouse model

For the OVX model, *Trim21*^+/+^ or *Trim21*^−/−^ mice (females; 12 weeks old) were randomly divided into two groups: the sham-operated group (*n* = 5) and the OVX group (*n* = 5). Bilateral ovariectomy was performed to induce osteoporotic bone loss under sodium pentobarbital-induced anesthesia in the OVX group, while in the sham-operated group, the ovaries were just exteriorized and then placed back. The incisions of the muscle and the skin were closed using 6-0 silk sutures. All of the mice were sacrificed 8 weeks after OVX modeling, and the mouse tibias were collected for micro-CT analysis, followed by the assessment of histological changes.

For the LPS model, *Trim21*^+/+^ or *Trim21*^−/−^ mice (males; ~12 weeks old) and *Trim21*^*f/f*^ or *Ctsk-cre; Trim21*^*f/f*^ mice (~12 weeks old) were randomly divided into two groups: the PBS group (*n* ≥ 3) and the LPS group (*n* ≥ 5). The mice in the latter group were treated with LPS (Beyotime, Cat# ST1470, China; 5 mg·kg^−1^) by intraperitoneal injection on Day 0 and Day 4, and then, the bone specimens were collected on Day 8 for micro-CT analysis as previously described with minor modifications.^49,50^

### Micro-CT analysis

After the mice were sacrificed, the right tibia or craniums were isolated and fixed with 4% PFA for 48 h and then maintained in 70% ethanol. High-resolution ex vivo micro-CT (SkyScan1176, Bruker micro-CT, Kontich, Belgium) was used for image acquisition, and the scans were then integrated into 2D images. The trabecular region from 0.1 mm to 1.0 mm below the tibia growth plate was reconstructed for 3D visualization, and cortical bones were scanned at mid-diaphysis of the tibia. Quantitative analyses of the morphometric parameters were conducted using the appropriate software package, and the following morphometric indices were analyzed: bone volume per tissue volume (BV/TV), trabecular thickness (Tb.Th), trabecular number (Tb.N), trabecular separation (Tb.Sp), cortical BV/TV (Ct.BV/TV) and cortical bone thickness (Ct.th).

### Von kossa and masson staining

Tibias were fixed with 4% PFA at room temperature for 48 h. Bones were dehydrated using a gradual series of ethanol (70%, 95%, and 100%), infiltrated, and embedded without decalcification in methyl methacrylate. von Kossa (Servicebio, Cat# G1043, China) and Masson (Servicebio, Cat# GP1032, China) staining were performed on tibial sections according to the manufacturer’s instructions. The proportion of positively stained areas in the total area was captured.

### Histological staining and histomorphometric analysis

For histological analysis, tissues were fixed with 4% PFA for 48 h and incubated in DEPC-EDTA solution for decalcification. Then, the specimens were embedded in paraffin and sectioned at 5 μm. Sections were then used for H&E (Leagene, DH0006) and Safranin O (S/O) staining according to the manufacturer’s instructions (Servicebio, Cat# GP1051, China). Fat cell density was quantified using ImageJ software (National Institutes of Health, Bethesda, USA) from 5 random fields (200 ×) of metaphysis for each sample. For dynamic histomorphometric analysis, mice were injected with calcein (Sigma, Cat# C0875, Germany) at a dose of 20 mg·kg^−1^ of body weight on Day 7 and Day 1 before being sacrificed. Tissues were fixed with 4% PFA for 48 h, and 9 μm-thick sections were prepared for double-labeling fluorescent analysis. The mineral apposition rate (MAR) was analyzed using a laser scanning confocal microscope (Leica Microsystems, Mannheim, Germany).

### Immunohistochemistry (IHC) and IF for tissues

The bone section was prepared as we previously described.^51^ Briefly, tibial bone sections were boiled in Tris-EDTA (pH 9) buffer solution to retrieve antigens, quenched with 3% hydrogen peroxide, and then treated with 0.5% Triton X-100. Next, 1% goat serum was used to block tissues at 37 °C for 30 min. For IHC staining, sections were treated with primary antibody against YAP1 (CST, Cat# 14074, USA, 1:200) overnight at 4 °C. Tissue sections were then washed with PBST, incubated with secondary antibody and visualized using a diaminobenzidine (DAB) kit (CST, Cat# 8059, USA). Cell nuclei were counterstained with hematoxylin. Images were taken under a light microscope (Nikon, Tokyo, Japan). For IF staining, the bone sections were treated with primary antibodies, including those against Sox9 (CST, Cat# 82630, USA, 1:100) and β-catenin (Abcam, Cat# AB32572, USA, 1:100). After PBST washes, tissue sections were incubated with a peroxidase-conjugated anti-fluorescein antibody (CST, Cat#4413, USA, 1:500). Antifade mounting medium with DAPI was used to mount the slides. The images were captured using a laser scan confocal microscope (Zeiss LSM 880, Germany) and analyzed by Image-Pro Plus software.

### Statistical analysis

All data are expressed as the mean ± standard deviation (SD) with sample sizes indicated in either the figures and/or legends. For comparisons between the two groups, statistical analyses were performed by Student’s *t* test. One-way ANOVA was used to compare the effects of more than two groups. A *P* value of <0.05 was considered statistically significant.

### Supplementary information

Revision-Supplementary Information-BR

## Data Availability

All the data support the figures, and the other findings are available upon reasonable request to the corresponding authors.
