# Supplementary material for: Trim21 depletion alleviates bone loss in osteoporosis via activation of YAP1/β-catenin signaling
Source: Bone Res. 2023 Oct 26;11:56. doi: 10.1038/s41413-023-00296-3 (PMC10603047; doi:10.1038/s41413-023-00296-3)

**Supplementary Information**

**Trim21 depletion alleviates bone loss in osteoporosis via activation of the YAP1/β-catenin signaling**

Ri-Xu Liu^1,7^, Rong-He Gu^2^, Zhi-Peng Li^1^, Zhi-Quan Hao^1^, Qin-Xiao Hu^1^, Zhen-Yan Li^1^, Xiao-Gang Wang^3^, Wang Tang^1^, Xiao-He Wang^1^, Yu-Kai Zeng^1^, Zhen-Wei Li^1^, Qiu Dong^1^, Xiao-Feng Zhu^4^, Di Chen^5^, Ke-Wei Zhao^6^, Rong-Hua Zhang^4🖂^, Zhen-Gang Zha^1🖂^, Huan-Tian Zhang^1🖂^

^1^Department of Bone and Joint Surgery, the First Affiliated Hospital of Jinan University; Key Laboratory of Regenerative Medicine of Ministry of Education, Jinan University, Guangzhou, Guangdong 510630, China;

^2^School of Basic Medical Sciences of Guangxi Medical University, the Fifth Affiliated Hospital of Guangxi Medical University, Nanning, Guangxi 530022, China;

^3^Key Laboratory of Big Data-Based Precision Medicine, School of Engineering Medicine, Beihang University, Beijing 100191, China;

^4^Guangdong Provincial Key Laboratory of Traditional Chinese Medicine Informatization, College of Pharmacy, Jinan University, Guangzhou, Guangdong 510630, China;

^5^Research Center for Computer-aided Drug Discovery, Shenzhen Institute of Advanced Technology, Chinese Academy of Sciences, Shenzhen 518005, China;

^6^Guangzhou Key Laboratory of Chinese Medicine Research on Prevention and Treatment of Osteoporosis, the Third Affiliated Hospital of Guangzhou University of Chinese Medicine, Guangzhou 510375, China;

^7^Department of Orthopedic and Spine Surgery, The First Affiliated Hospital of Guangzhou Medical University, Guangzhou, Guangdong 510120, China

Correspondence: Rong-Hua Zhang ([tzrh@jnu.edu.cn](mailto:tzrh@jnu.edu.cn)) or Zhen-Gang Zha ([tbri@jnu.edu.cn](mailto:tbri@jnu.edu.cn)) or Huan-Tian Zhang ([zhanghuantian@jnu.edu.cn](mailto:zhanghuantian@jnu.edu.cn))

These authors contributed equally: Ri-Xu Liu, Rong-He Gu, Zhi-Peng Li, Zhi-Quan Hao

**Fig. S1** The correlation between *Trim21* expression and osteoporotic status

**Fig. S2** Establishment of *Trim21^-/-^* knockout and *Ctsk-cre; Trim21^f/f^* conditional knockout mice.

**Fig. S3** The effect of Trim21 on the length of long bones and cortical bone parameters

**Fig. S4** mBMSCs verification and osteogenic differentiation upon loss of Trim21

**Fig. S5** The role of Trim21 in osteogenic differentiation and bone repair

**Fig. S6** The role of Trim21 in regulating OCs formation

**Fig. S7** Involvement of the YAP1/BCL9/β-catenin complex in *Trim21*-regulated osteoblast differentiation

**Fig. S8** *Trim21* depletion alleviates LPS-induced bone loss.

**Fig. S9** *Trim21* depletion alleviates OVX-induced bone loss through β-catenin signaling.

**Table S1** A detailed information of each patient enrolled for analysis

**Table S2** The primers used for Trim21 global and conditional knockout mice genotyping

**Table S3** List of the siRNAs sequences

**Table S4** The primers used for quantitative RT-PCR

**Table S5** Methods used for the determination of indicated phenotypes.


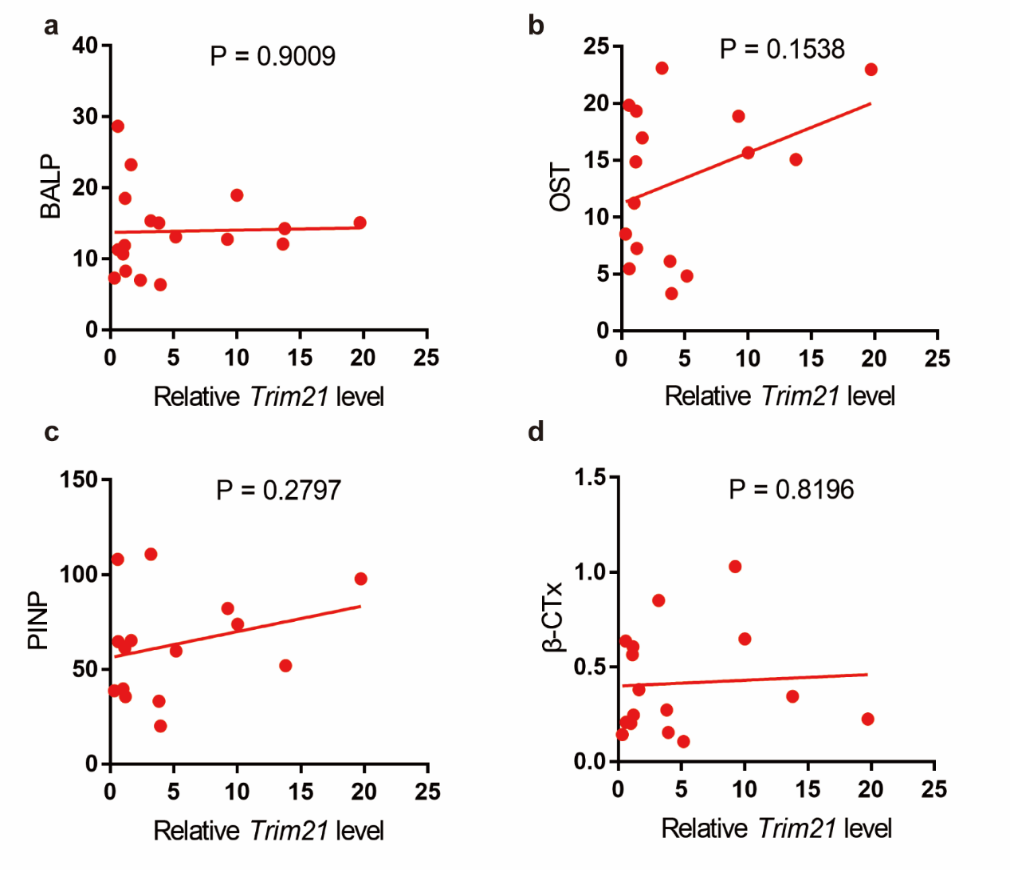


**Fig. S1** The correlation between *Trim21* expression and osteoporotic status. **a-d** Correlation analysis between the mRNA expression of *Trim21* with bone alkaline phosphatase (BALP), amino-terminal propeptide of type I collagen (PINP), osteocalcin (OST), and C-terminal telopeptide of type I collagen (β-CTx) in patients with different bone mineral densities (BMD).

**
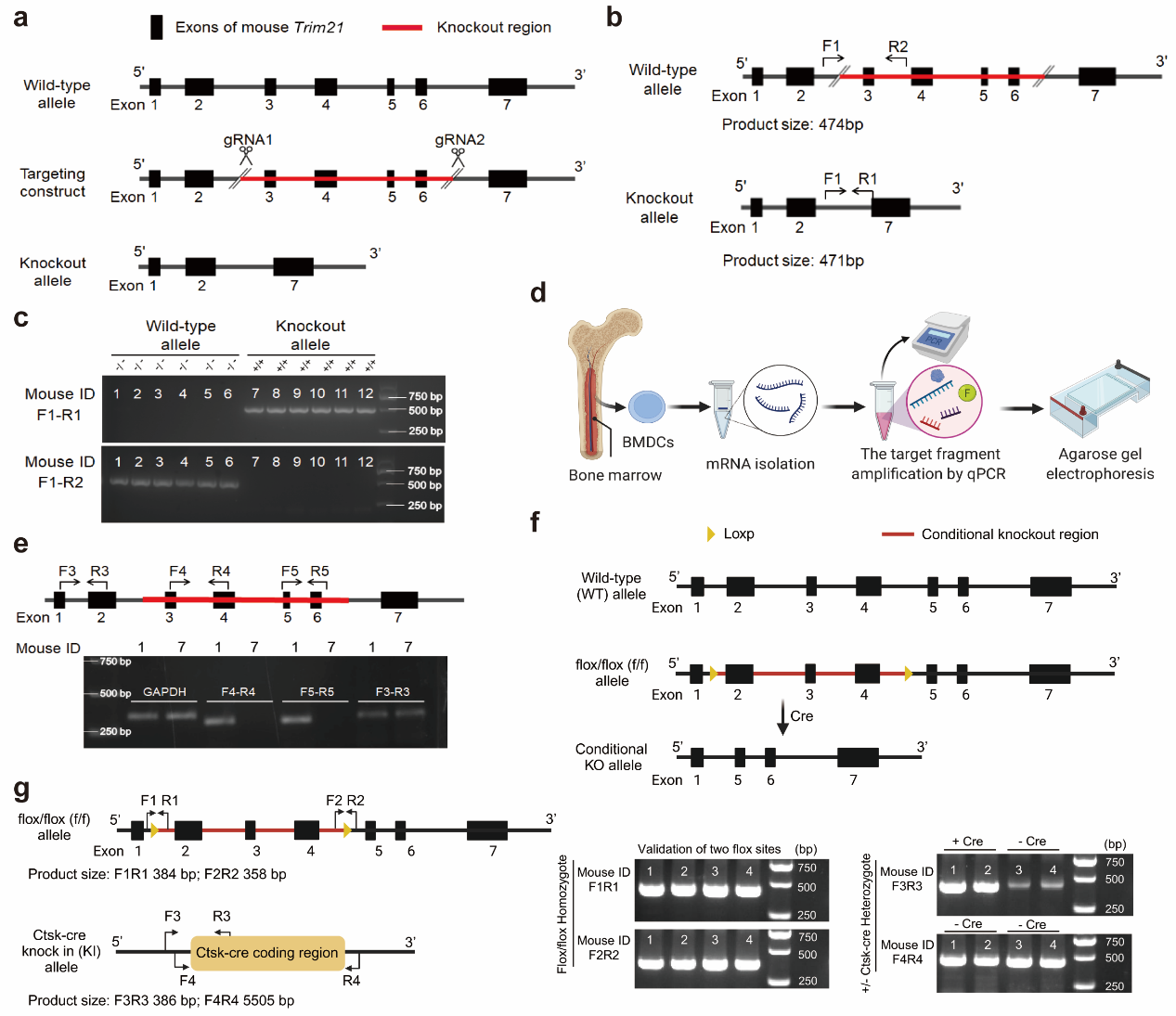
**

**Fig. S2** Establishment of *Trim21^-/-^* knockout and *Ctsk-cre; Trim21^f/f^* conditional knockout mice. **a** *Trim21* global knockout (*Trim21*^-/-^) mice were designed and generated. **b, c** *Trim21^+/+^* (Wild-type) and *Trim21^-/-^* (Knockout) mice were validated via genotyping by Reverse Transcription Polymerase Chain Reaction (RT-PCR). d, e Genotyping verification of *Trim21^+/+^* and *Trim21^-/-^* mice by qRT-PCR with multiple primer pairs. The primers used for RT-PCR were: forward (F3): 5′-CCTGGTTAGATTCCACGGCA-3′, reverse (R3): 5′-GGACATGAACTGCCCCCATT-3′; forward (F4): 5′-GCTGGAGAGGAGGATTCGTG-3′, reverse (R4): 5′-CCCATCCTCACTTGTCTCCG-3′; forward (F5): 5′-GGGAGACACCCATCAGAACG-3′, reverse (R5): 5′-AGGAGAATCTCTGGGCACCT-3′. Mouse ID-1 in the blot indicated *Trim21^-/-^* mice, while Mouse ID-7 in the blot indicated *Trim21^+/+^*mice. **f** *Trim21* conditional knockout (*Trim21^f/f^*) mice were designed and generated. **g** Genotyping verification of *Trim21^f/f^* and *Ctsk-cre; Trim21^f/f^* mice by RT-PCR.


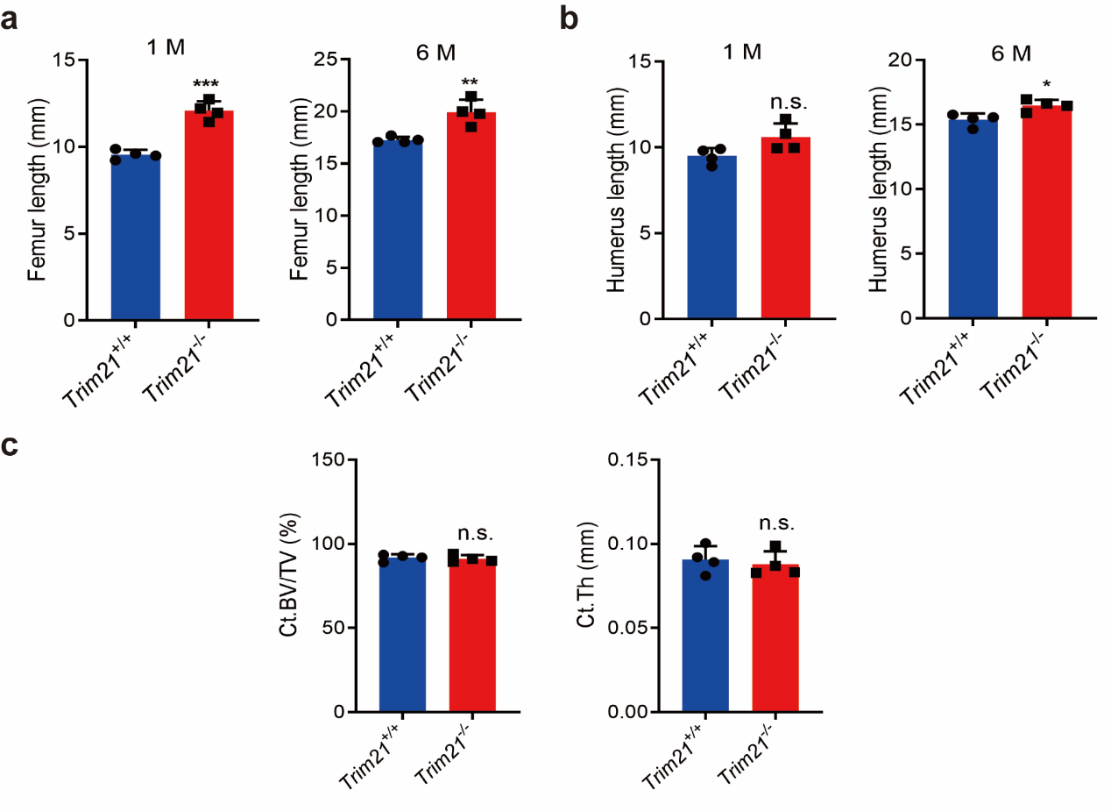


**Fig. S3** The effect of Trim21 on the length of long bones and cortical bone parameters. **a, b** Quantitative analysis of the femur and humerus length of mice at 1-month-old (1 M) and 6-month-old (6 M). **c** Cortical bone volume per tissue volume (Ct. BV/TV) and cortical thickness (Ct. Th) of the proximal tibia in the 14-week-old *Trim21^+/+^* and *Trim21^-/-^* mice. All bar graphs are presented as mean ± SD. **P*< 0.05; ***P*< 0.01; ****P*< 0.001; n.s., not significant; unpaired Student’s *t*-test.

**
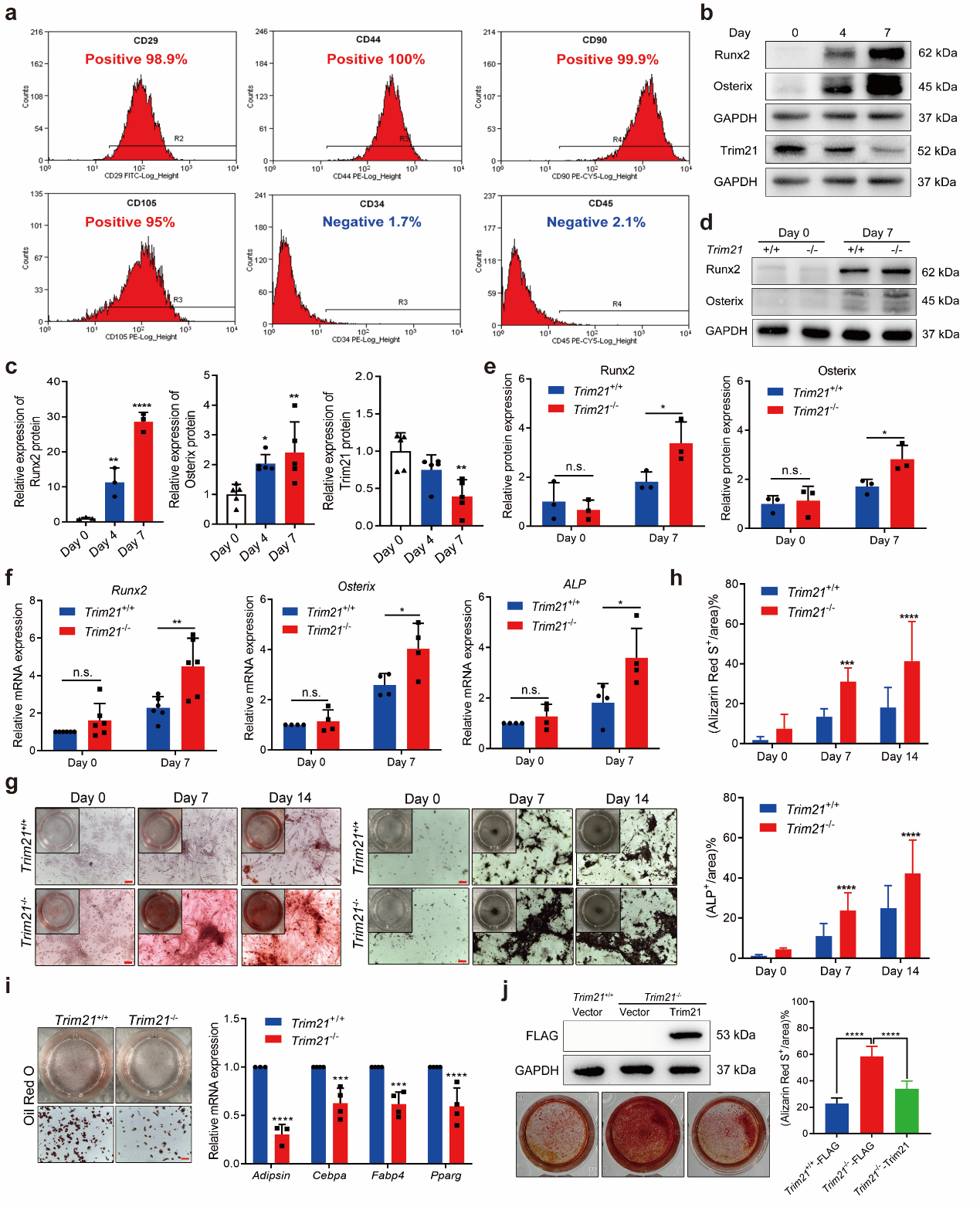
Fig. S4** BMSCs verification and osteogenic differentiation upon loss of Trim21*.* **a** Flow cytometry detection of mouse BMSCs positive markers (CD29, CD44, CD90, and CD105) and negative markers (CD34 and CD45). **b, c** Immunoblotting analysis and quantification of Runx2, Osterix, and Trim21 expression in the mouse BMSCs with or without osteogenic induction. **d, e** Immunoblotting analysis and quantification of Runx2 and Osterix expression in the BMSCs isolated from *Trim21^+/+^* and *Trim21^-/-^* mice with or without osteogenic induction. **f** Quantitative RT-PCR analysis of osteogenic biomarker genes (*Runx2, Osterix, and ALP*) in the BMSCs isolated from the indicated mice with or without osteogenic induction. **g** Alizarin Red S and ALP staining of BMSCs derived from *Trim21^+/+^* and *Trim21^-/-^* mice after induction with osteogenic medium for different times. Scale bar: 50 μm. **h** Histogram showing Alizarin Red S- (n ≥ 3) and ALP- (n ≥ 3) staining quantification. **i** Oil Red O staining of mouse BMSCs after 7 days of adipogenic induction (left panel, scale bar = 50 μm). Quantitative RT-PCR detection of adipogenic biomarker genes (*Adipsin, Cebpa, Fabp4, and Pparg*) in mouse BMSCs with adipogenic induction (right panel). **j** Immunoblotting analysis of FLAG-Vector and FLAG-Trim21 expression in the mouse BMSCs during osteogenic induction (upper panel). Representative images of Alizarin Red S staining of the mouse BMSCs in different groups (lower panel). Quantitative analysis of the percentage of Alizarin red S-stained (n ≥ 3) area (right panel). All bar graphs are presented as mean ± SD. **P*< 0.05; ***P*< 0.01; ****P*< 0.001; *****P*< 0.0001; n.s., not significant by Student’s *t*-test.


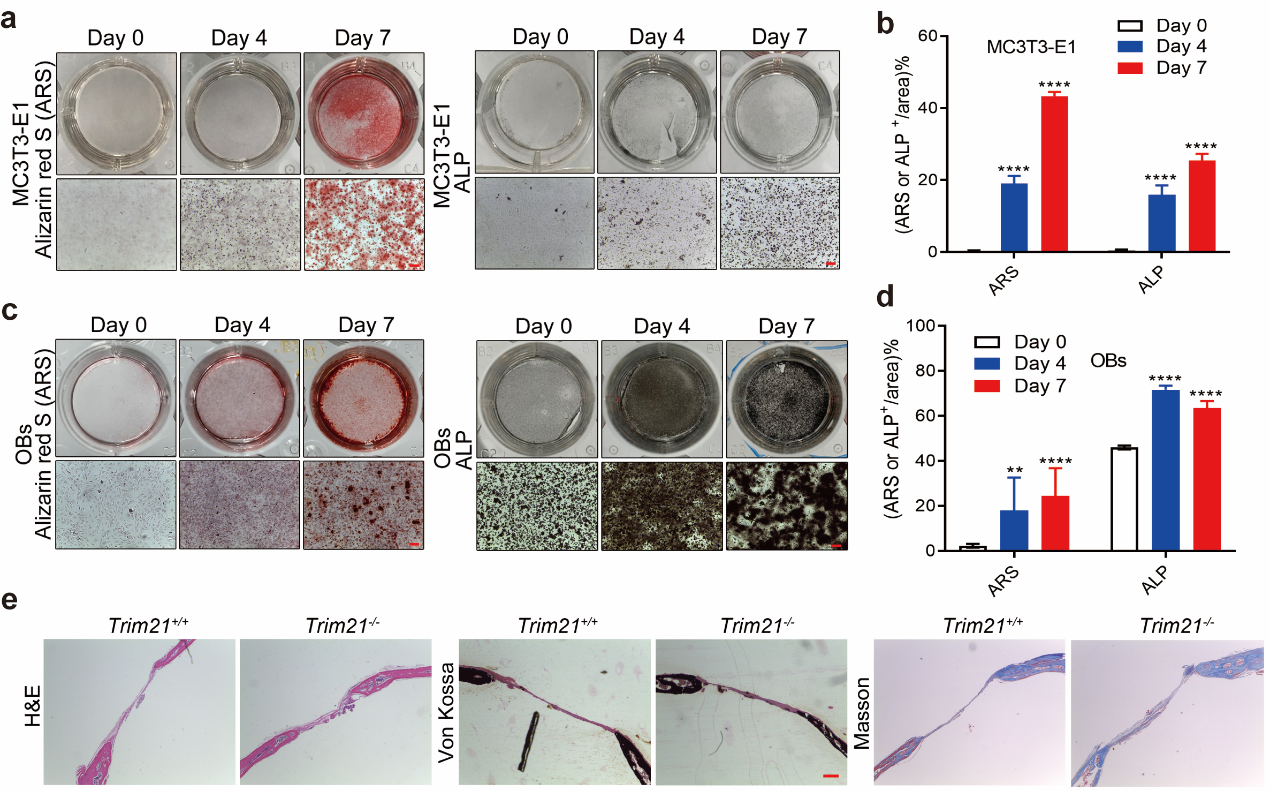


**Fig. S5** The role of Trim21 in osteogenic differentiation and bone repair. **a, b** Alizarin red S (ARS, left panel) or ALP (right panel) staining of MC3T3-E1 after induction with osteogenic medium for different times (**a**). Scale bar = 50 μm. The percentage of ARS- (n ≥ 3) and ALP-stained (n ≥ 3) area (**b**). **c, d** ARS (left panel) and ALP (right panel) staining of primary OBs after induction with osteogenic medium for different times (**c**). Scale bar: 50 μm. The percentage of ARS- (n ≥ 3) and ALP-stained (n ≥ 3) area (**d**). **e** Representative images of H&E staining, von Kossa staining, and Masson staining of calvarial bone defect of 2-month-old *Trim21^+/+^* and *Trim21^-/-^* mice after surgical induction for 1 month. Scale bar: 500 μm. All bar graphs are presented as mean ± SD. ***P*< 0.01; *****P*< 0.0001 by one-way ANOVA followed by Tukey post hoc test.

**
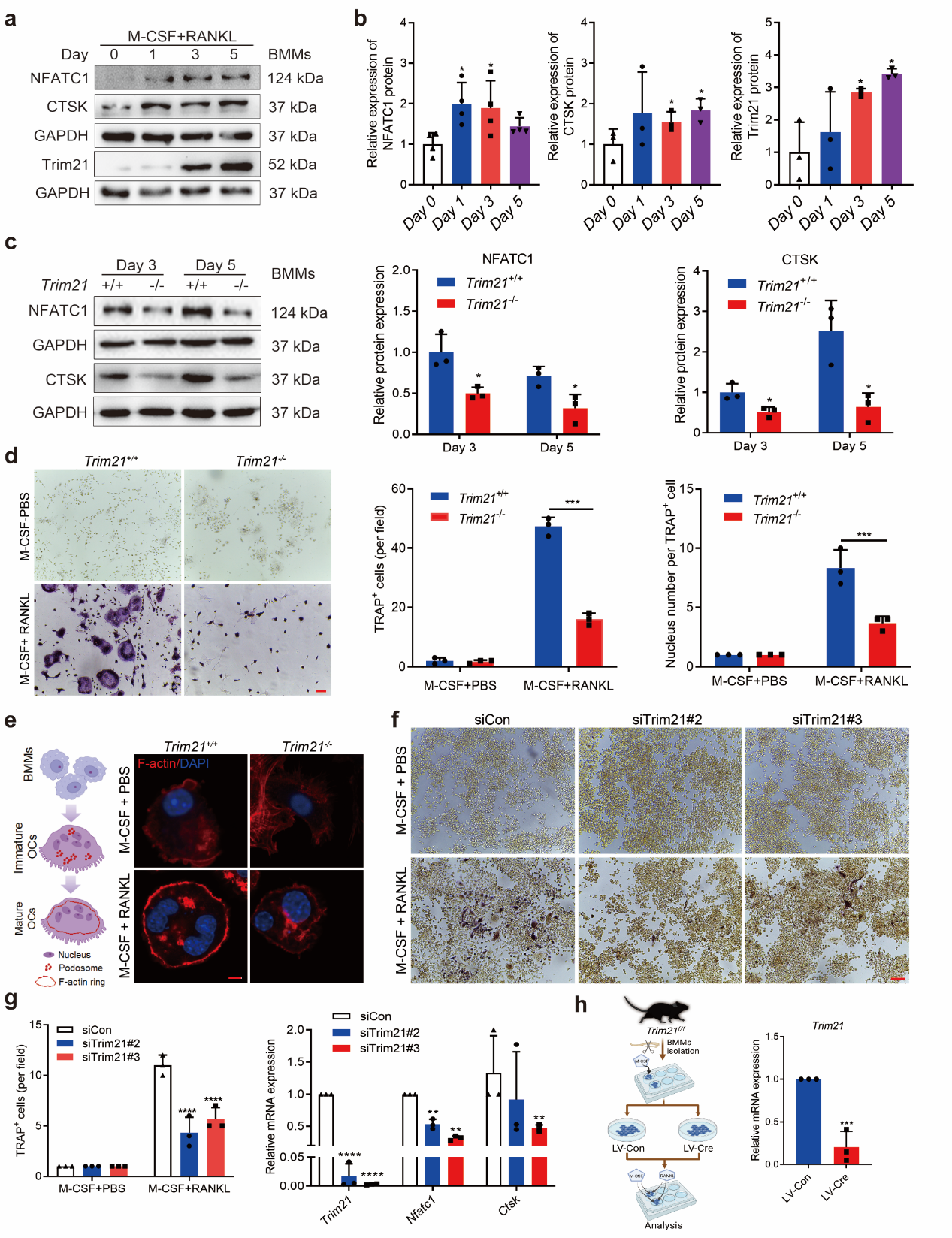
Fig. S6** The role of Trim21 in regulating OCs formation. **a, b** Immunoblotting analysis of NFATC1, CTSK, and Trim21 expression in bone marrow macrophages (BMMs) upon induction with 30 ng/ml M-CSF and 100 ng/ml RANKL for 0, 1, 3, and 5 days (**a**). Quantification of relative protein expression levels of Trim21, NFATC1, and CTSK of Immunoblotting (**b**). **c** Immunoblotting analysis of osteoclastogenesis makers (NFATC1 and CTSK) and relative quantification of expression levels. BMMs derived from 6-week-old *Trim21^+/+^* and *Trim21^-/-^* mice were induced by 30 ng/ml M-CSF and 100 ng/ml RANKL for 3 days or 5 days. **d**, Representative images (left panel) in BMMs derived from 6-week-old *Trim21^+/+^* and *Trim21^-/-^* mice were induced for osteoclastogenesis for 5 days. Scale bar: 50 μm. Quantification of TRAP stained-positive OCs and nucleus number per TRAP^+^ cell (right panel). **e** Schematic diagram illustrating the F-actin ring formation in BMMs-derived OCs (left panel). Immunofluorescence staining of F-actin (red) and DAPI (blue) in BMMs-derived OCs. Scale bar: 20 μm. **f, g** Representative images (**f**), quantification data of TRAP stained-positive OCs (**g**), and quantitative RT-PCR detection of OCs markers (**g**), in the induction of osteoclast differentiation of RAW264.7 cells with 30 ng/ml M-CSF and 100 ng/ml RANKL for 5 days. For the knockdown of the Trim21 expression, the siTrim21#2 and siTrim21#3 siRNAs were applied. Scale bar: 50 μm. **h** Schematic diagram illustrates the osteoclastogenesis induction of BMMs derived from *Trim21^f/f^* mice infected with control lentivirus (LV-Con) or lentivirus expressing Cre (LV-Cre). All bar graphs are presented as mean ± SD. * *p*< 0.05; ** *p*< 0.01; *** *p*< 0.001; **** *p*< 0.0001; n.s., not significant by Student’s *t*-test.

**
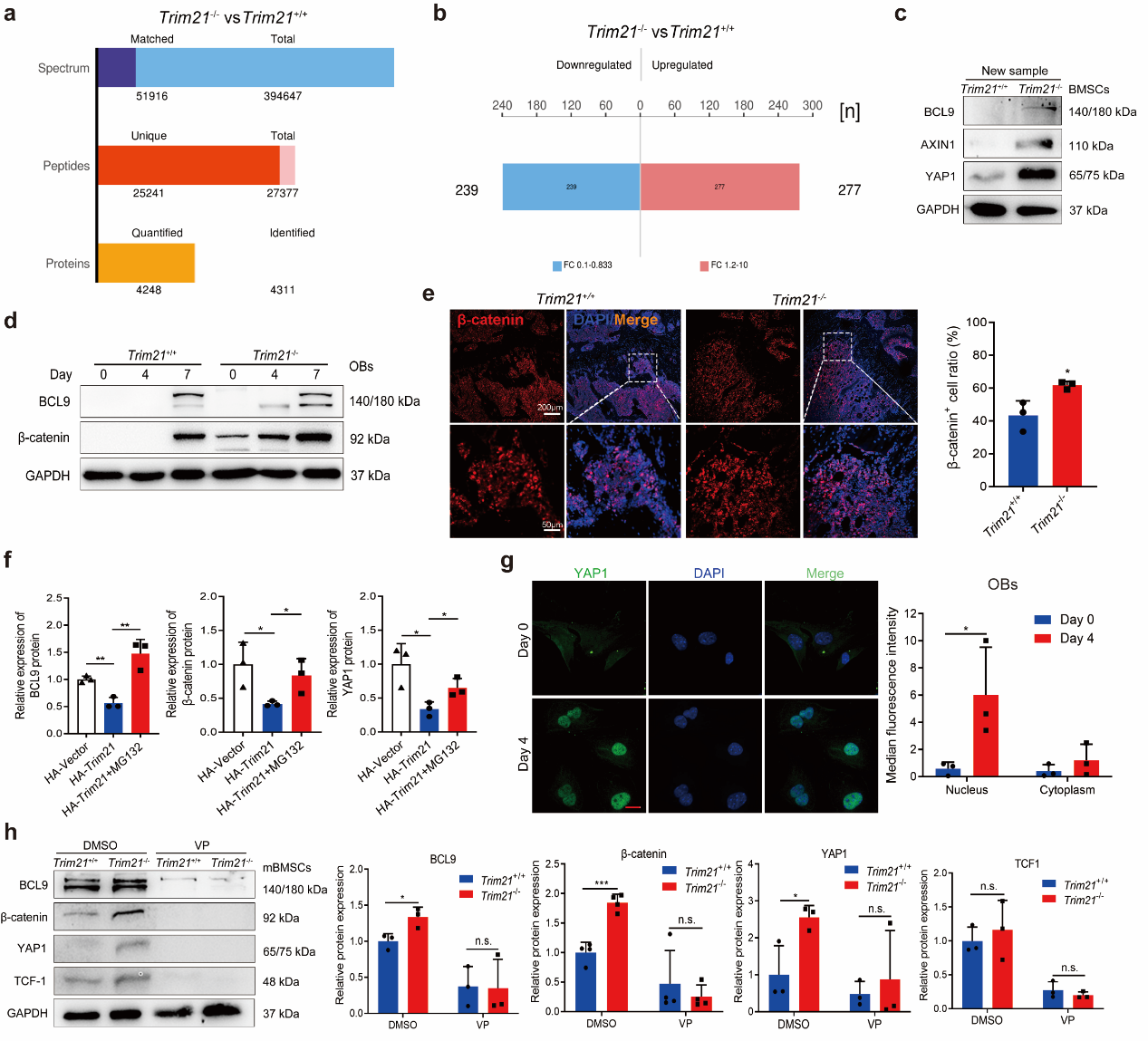
**

**Fig. S7** Involvement of the YAP1/BCL9/β-catenin complex in *Trim21*-regulated osteoblast differentiation*.* **a** Identification number of spectra, peptides, and proteins in *Trim21*^+/+^ and *Trim21*^-/-^ mouse BMSCs analyzed by TMT-based quantitative proteomics. **b** Schematic diagram illustrating the downregulated (*Trim21*^-/-^: *Trim21*^+/+^ < 0.8, p < 0.05) and upregulated (*Trim21*^-/-^: *Trim21*^+/+^ > 1.2, p < 0.05) differentially expressed proteins (DEPs) identified in the BMSCs of *Trim21^+/+^* and *Trim21^-/-^* mice. **c** Immunoblotting analysis of BCL9, AXIN1, and YAP1 protein expression in the mouse BMSCs. New sample: the newly prepared mouse BMSCs protein samples of the two groups. **d** Immunoblotting analysis of BCL9 and β-catenin expression in the OBs derived from *Trim21^-/-^* and *Trim21^+/+^* mice with or without osteogenic induction for 0, 4, and 7 days. **e** Representative immunofluorescence images and quantitative analysis of β-catenin/DAPI staining of the tibia sections in 1-month-old *Trim21*^+/+^ and *Trim21*^-/-^ mice. Scale bar: 50 μm. **f** Immunoblotting quantification of BCL9, β-catenin, and YAP1 protein expression in the HEK293T treated with or without MG132. **g** Immunofluorescence staining for subcellular localization of YAP1 in OBs during osteogenic induction at different times (left panel). Scale bar: 50 μm. Quantitative analysis of median fluorescence intensity of the nuclear and cytoplasmic YAP1 in OBs (right panel). **h** Immunoblotting analysis and quantification of BCL9, β-catenin, YAP1, and TCF-1 protein expression in the BMSCs isolated from different mice with or without treatment of Verteporfin (VP), followed by the osteogenic induction. All bar graphs are presented as mean ± SD. **P*< 0.05; ** *p*< 0.01; ****P*< 0.001; n.s., not significant by Student’s *t*-test.

**
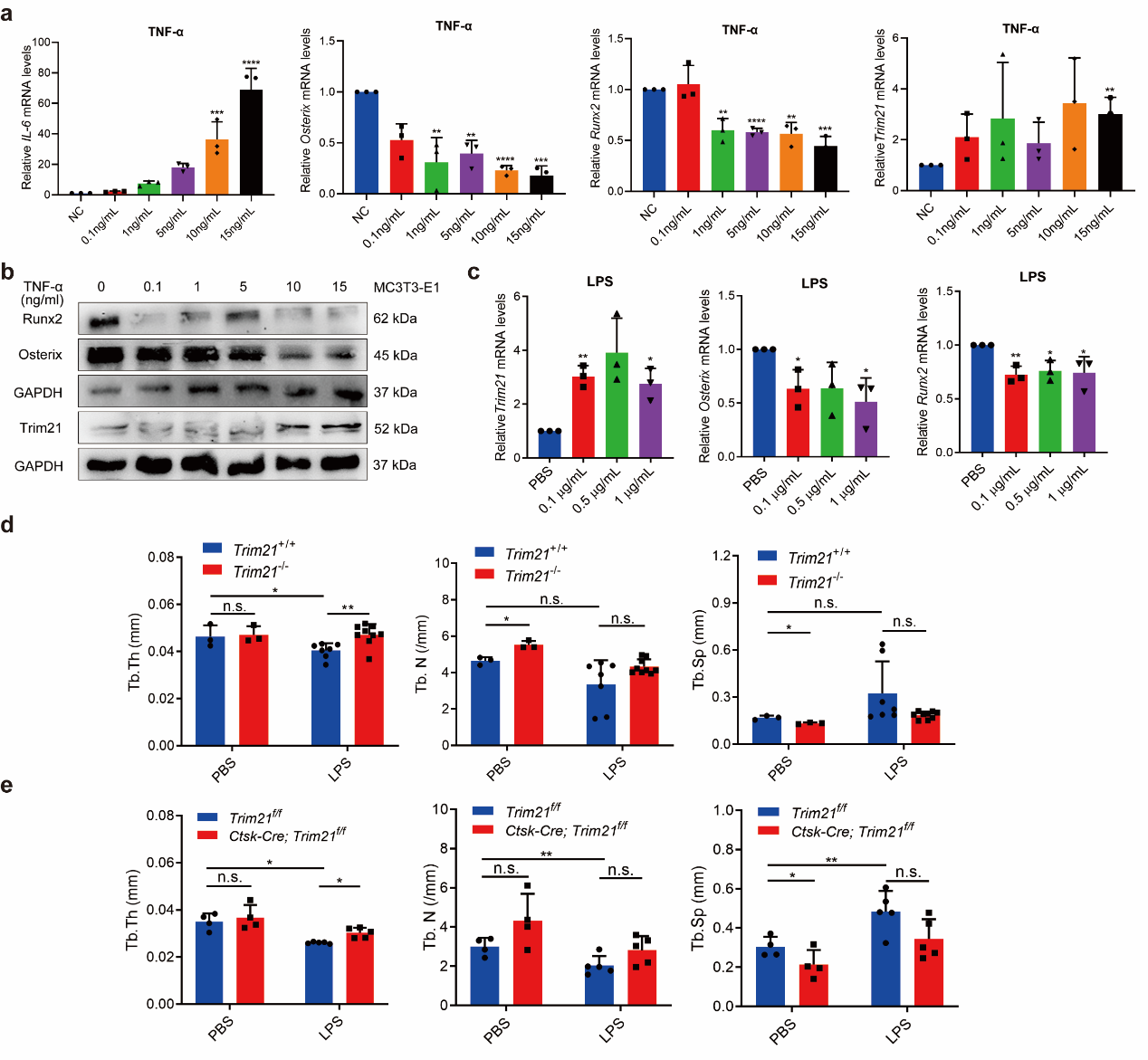
**

**Fig. S8** *Trim21* depletion alleviates LPS-induced bone loss. **a** Quantitative RT-PCR analysis of *IL-6*, *Trim21*, *Runx2*, and *Osterix* mRNA expression in the MC3T3-E1 cells treated with different concentrations of TNF-α for 24 h. **b** Immunoblotting analysis of Runx2, Osterix, and Trim21 expression in the MC3T3-E1 cells treated with different concentrations of TNF-α for 24 h. **c** Quantitative RT-PCR analysis of *Trim21*, *Runx2*, and *Osterix* mRNA expression in the MC3T3-E1 cells treated with different concentrations of LPS for 24 h. **d** Micro-CT analysis (Tb. Th, Tb. N, and Tb. Sp) of proximal tibia bone of the 12-week-old *Trim21^+/+^* and *Trim21*^-/-^ mice induced by either LPS or control PBS for 8 days. **e** Micro-CT analysis (Tb. Th, Tb. N, and Tb. Sp) of proximal tibia bone of the 12-week-old *Trim21^f/f^* and *Ctsk-Cre; Trim21^f/f^* mice induced by LPS or control PBS for 8 days. All bar graphs are presented as mean ± SD. **P*< 0.05; ***P*< 0.01; ****P*< 0.001; *****P*< 0.0001; n.s., not significant by paired Student’s *t*-test.


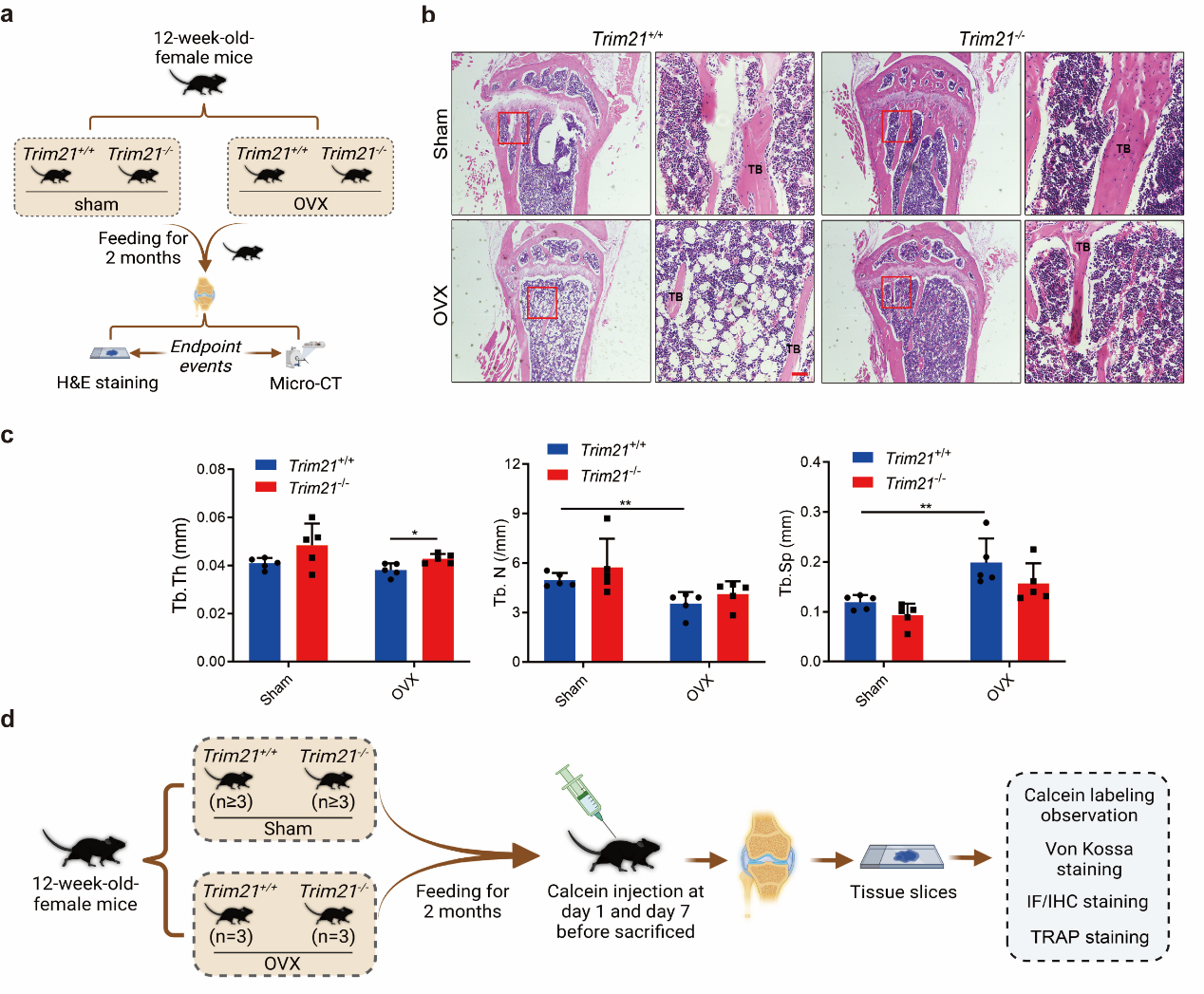


**Fig. S9** *Trim21* depletion alleviates OVX-induced bone loss through β-catenin signaling. **a** Schematic diagram showing the H&E staining and micro-CT analysis of the proximal tibia in *Trim21*^+/+^ and *Trim21*^-/-^ mice induced by sham-operated or OVX. **b** Representative H&E staining images of tibia sections showing the marrow adipose tissue. The regions in the red square inset were enlarged in the corresponding right panel. Scale bar: 50 μm. **c** Micro-CT analysis (Tb. Th, Tb. N, and Tb. Sp) of the proximal tibia bone of *Trim21^+/+^* and *Trim21*^-/-^ mice induced by sham-operated or OVX. **d** Schematic diagram showing the histological analysis (Calcein labeling, von Kossa, IF/IHC, and TRAP staining) of the proximal tibia in *Trim21*^+/+^ and *Trim21*^-/-^ mice induced by sham-operated or OVX. All bar graphs are presented as mean ± SD. **P*< 0.05; ***P*< 0.01; n.s., not significant; Student’s *t*-test.

**Table S2. The primers used for Trim21 global and conditional knockout mice genotyping**

| **siRNA** | **Forward** | **Reverse** |
| --- | --- | --- |
| *Trim21^-/-^*  *(F1, R1)* | TCCCTATTGAAGAGGCTGCTAAG | TTGAGATGATGAGCCATGAGTTG |
| *Trim21^-/-^*  *(F1, R2)* | TCCCTATTGAAGAGGCTGCTAAG | CCTACAGCTCTTGCCTTCCAGTC |
| *Trim21^f/f^*  *(F1, R1)* | ATATGGGCACGCCTACATTGATG | GCATTAGGAGTCAAGGTTTATGCAGC |
| *Trim21^f/f^*  *(F2, R2)* | TCATACCTGTGTCACTGTCTCAGTCAG | AAGCTTTGTCCTGTGCTGTGGCTAAT |
| *Trim21^f/f^*  *(F3, R3)* | GGGCAGTCTGGTACTTCCAAGCT | GAGCCCAGATCCACATCTGAACTG |
| *Trim21^f/f^*  *(F4, R4)* | CAGCAAAACCTGGCTGTGGATC | ATGAGCCACCATGTGGGTGTC |

**Table S3. List of the siRNAs sequences**

| **siRNA** | **Forward** | **Reverse** |
| --- | --- | --- |
| *siTrim21#2* | GGAACAUUGACACCCAGAATT | UUCUGGGUGUCAAUGUUCCTT |
| *siTrim21#3* | GCGUUGUCUCCUUCUACAATT | UUGUAGAAGGAGACAACGCTT |

**Table S4. The primers used for quantitative RT-PCR**

| **Genes** | **Forward** | **Reverse** |
| --- | --- | --- |
| **Human** |  |  |
| *h-TRIM21* | CCAATCCGTGGCTGATACTT | GCACCCAGGACCATAGGATA |
| *h-GAPDH* | CTGACTTCAACAGCGACACC | CCCTGTTGCTGTAGCCAAAT |
| **Mice** |  |  |
| *GAPDH* | TGTGTCCGTCGTGGATCTGA | TTGCTGTTGAAGTCGCAGGAG |
| *Trim21* | TGGAGAGGAGGATTCGTGGT | AGGGCATGTGCTTGTTAGGT |
| *Runx2* | CACTACCCAGCCACCTTTAC | AGGATGCTGACGAAGTACCA |
| *Osterix* | CCAGGCAACACACCTACTCC | GGGAGCAAAGTCAGATGGGT |
| *ALP* | TGGACGGTGAACGGGAAAAT | CATACGCCATCACATGGGGA |
| *Adipsin* | GACGGATGACGACTCTGTGC | GGTGAGGCACTACACTCTGC |
| *Cebpa* | TTCGGGTCGCTGGATCTCTA | TCAAGGAGAAACCACCACGG |
| *Fabp4* | CGACAGGAAGGTGAAGAGCAT | AACACATTCCACCACCAGCTT |
| *Pparg* | GCCAAGGTGCTCCAGAAGAT | GGGTGAAGGCTCATGTCTGT |
| *NFATC1* | TTCCGAGTTCACATCCCACA | AGTCTTTGCTTCCATCTCCCA |
| *Ctsk* | CAGTAGCCACGCTTCCTATCC | ACGCCGAGAGATTTCATCCA |
| *ACP5* | CAAAGAGATCGCCAGAACCG | ACGTCCTCAAAGGTCTCCTG |
| *Atp6v0d2* | CCTGGTTCGAGGATGCAAAG | GGTCTCACACTGCACTAGGT |
| *MMP9* | CCAGCCGACTTTTGTGGTCTT | CGGGTGTAACCATAGCGGT |
| *IL-6* | GGCTAAGGACCAAGACCATCC | GCACTAGGTTTGCCGAGTAGA |
| OCN | GAACAGACAAGTCCCACACAGC | TCAGCAGAGTGAGCAGAAAGAT |
| OPG | GCCACGCAAAAGTGTGGAAT | TTTGGTCCCAGGCAAACTGT |
| RANKL | GAGCACGAAAAACTGGTCGG | AGGGTTGGACACCTGAATGC |

**Table S5. Methods used for the determination of indicated phenotypes**


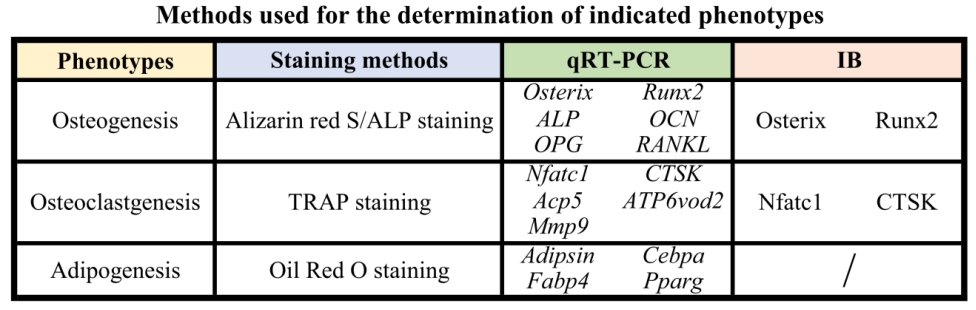

Supplement: Supplementary file 1 — Revision-Supplementary Information-BR [file 41413_2023_296_MOESM1_ESM.docx]
